# Supplementary figures and images for: Oligodendrocytes support axonal transport and maintenance via exosome secretion
Source: PLoS Biol. 2020 Dec 22;18(12):e3000621. doi: 10.1371/journal.pbio.3000621 (PMC7787684; doi:10.1371/journal.pbio.3000621)

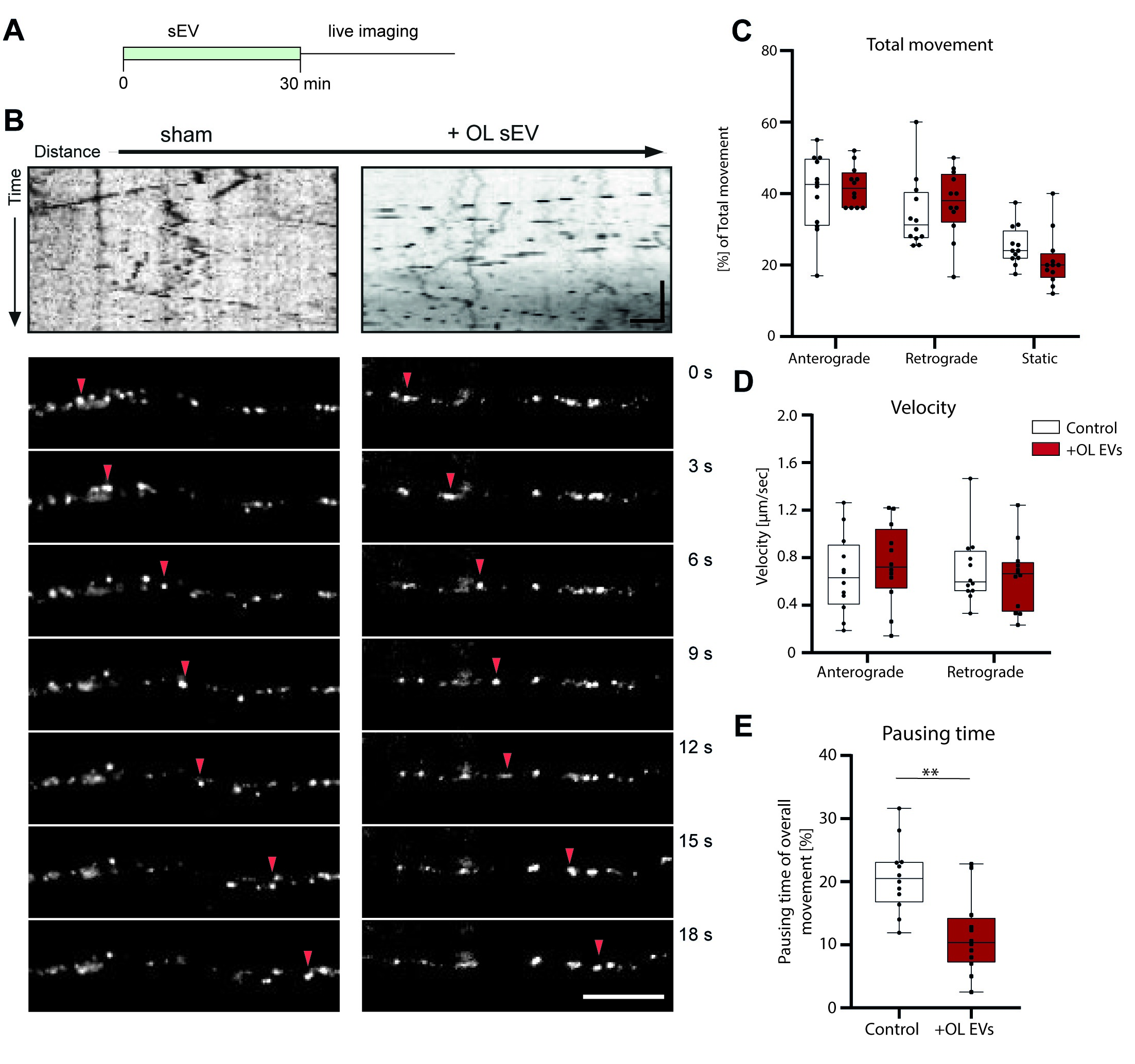

Supplement: S1 Fig — (A) Illustration of experimental schedule. sEVs were applied to hippocampal neurons and imaged after 30 min. (B) Representative kymographs and corresponding time-lapse frames illustrating movement of BDNF-mCherry-positive vesicles along the axon of primary hippocampal neurons. Neurons were sham treated (control) or treated with oligodendroglial (OL) sEVs. Red arrowhead follows 1 distinct particle moving over time. Kymograph: horizontal scale bar = 5 μm, vertical scale bar = 1 min; frames: scale bar = 5 μm. (C–E) Quantitative analysis of kymographs considering total movement (C), velocity (D), and pausing time (E) of BDNF-mCherry-positive vesicles. Data are presented as median, boxes (25th percentile to 75th percentile), and whiskers (minimum to maxamium showing all data points), n = 12 recorded neurons from 3 independent experiments. **p < 0.01, Shapiro–Wilk normality test and 2-tailed, non-parametric, unpaired Mann–Whitney test. Underlying data can be found in S1 Data. (TIF) [file pbio.3000621.s003.tif]

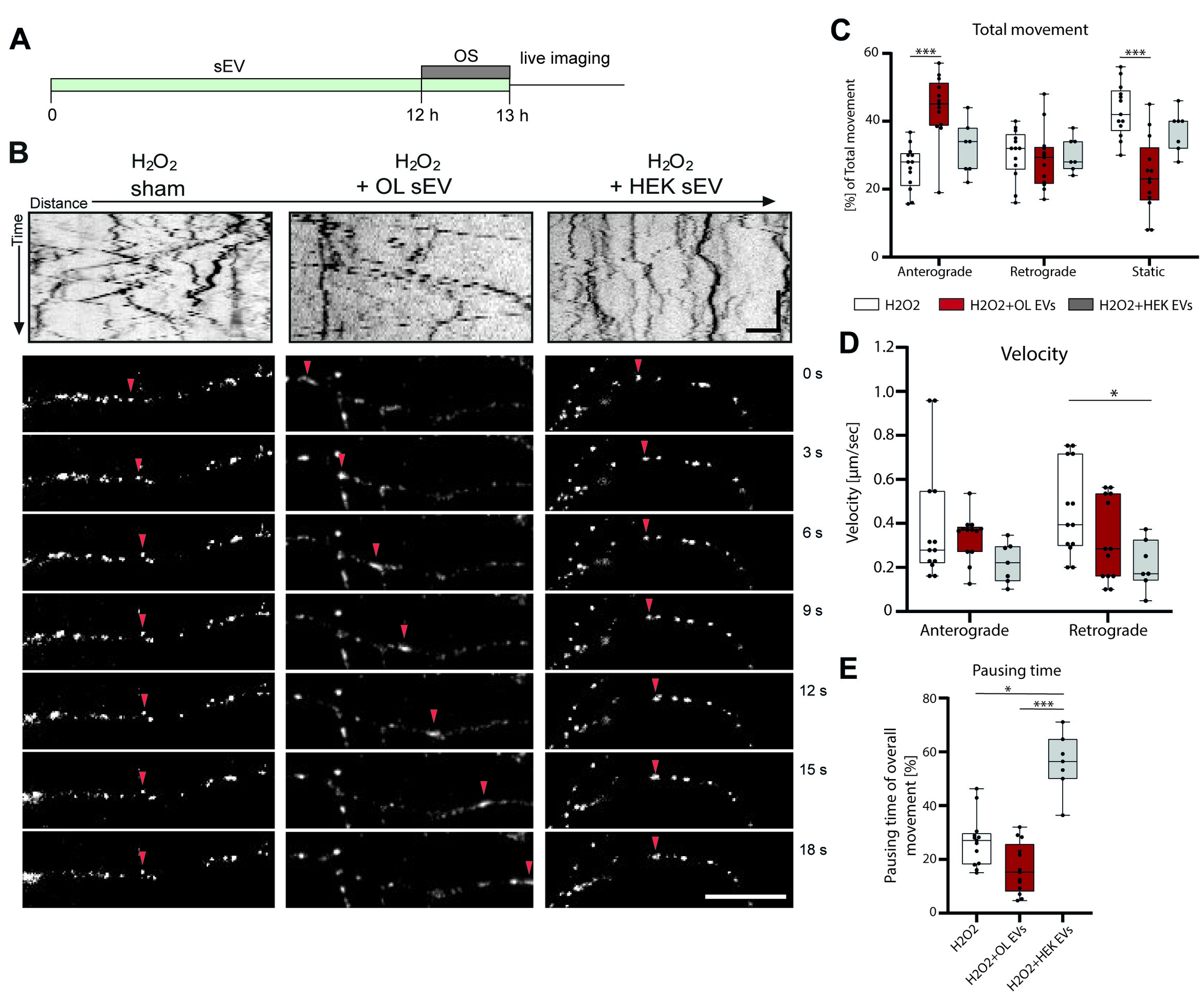

Supplement: S2 Fig — (A) Illustration of experimental schedule. Hippocampal neurons were pretreated with sEVs before exposure to oxidative stress (OS) and live imaging. (B) Representative kymographs and corresponding time-lapse frames illustrating movement of BDNF-mCherry-positive vesicles generated from hippocampal neurons sham treated or treated with sEVs derived from oligodendrocytes (OL) or HEK293T cells. Red arrowhead follows 1 distinct particle moving over time. Kymograph: horizontal scale bar = 5 μm, vertical scale bar = 1 min; frames: scale bar = 5 μm. (C–E) Quantitative analysis of kymographs regarding total movement (C), velocity (D), and pausing time (E) of BDNF-mCherry-positive vesicles. Data are presented as median, boxes (25th percentile to 75th percentile), and whiskers (minimum to maxamium showing all data points), n = 13 for untreated and OL-sEV-treated neurons and n = 7 for HEK-sEV-treated neurons derived from 3 independent experiments. *p < 0.05, **p < 0.01, ***p < 0.001, Shapiro–Wilk normality test following non-parametric, unpaired Kruskal–Wallis test with Dunn’s multiple comparison test. Underlying data can be found in S1 Data. (TIF) [file pbio.3000621.s004.tif]

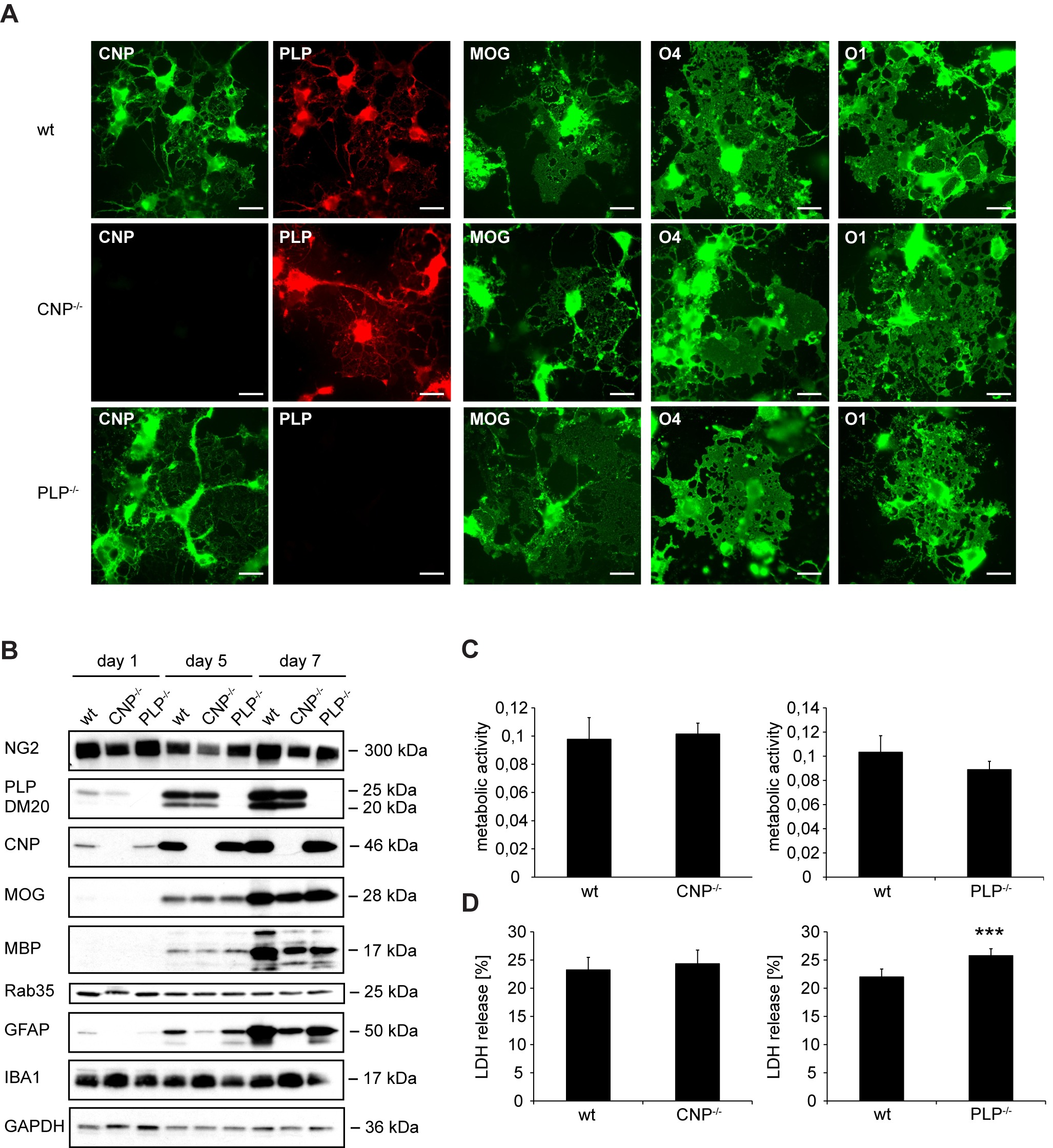

Supplement: S3 Fig — Wild-type, PLP-null, and CNP-null oligodendrocytes were compared regarding their differentiation and cell viability. (A) Immunofluorescence staining of primary cultured oligodendrocytes after 6 days in vitro (DIV) using antibodies against PLP and CNP as well as differentiation markers MOG, O4, and O1. Morphological appearance and differentiation of mutant oligodendrocytes appear normal. (B) Western blot analysis of wild-type and mutant oligodendrocytes after 1, 5, and 7 DIV using differentiation markers (NG2, PLP/DM20, CNP, MOG, MBP) as well as Rab35, which is a GTPase regulating sEV/exosome release. GAPDH serves as loading control. (C) MTT cell viability assay conducted at 6 DIV, n = 8. (D) Lactate dehydrogenase (LDH) cytotoxicity assay to determine cell death in the cultures. At day 6 in culture, primary oligodendrocytes were incubated with fresh medium for 24 h and subjected to LDH assay (Roche, carried out according to the manufacturer’s protocol), n = 8. Underlying images of blots and data can be found in S1 Images and S1 Data, respectively. (TIF) [file pbio.3000621.s005.tif]

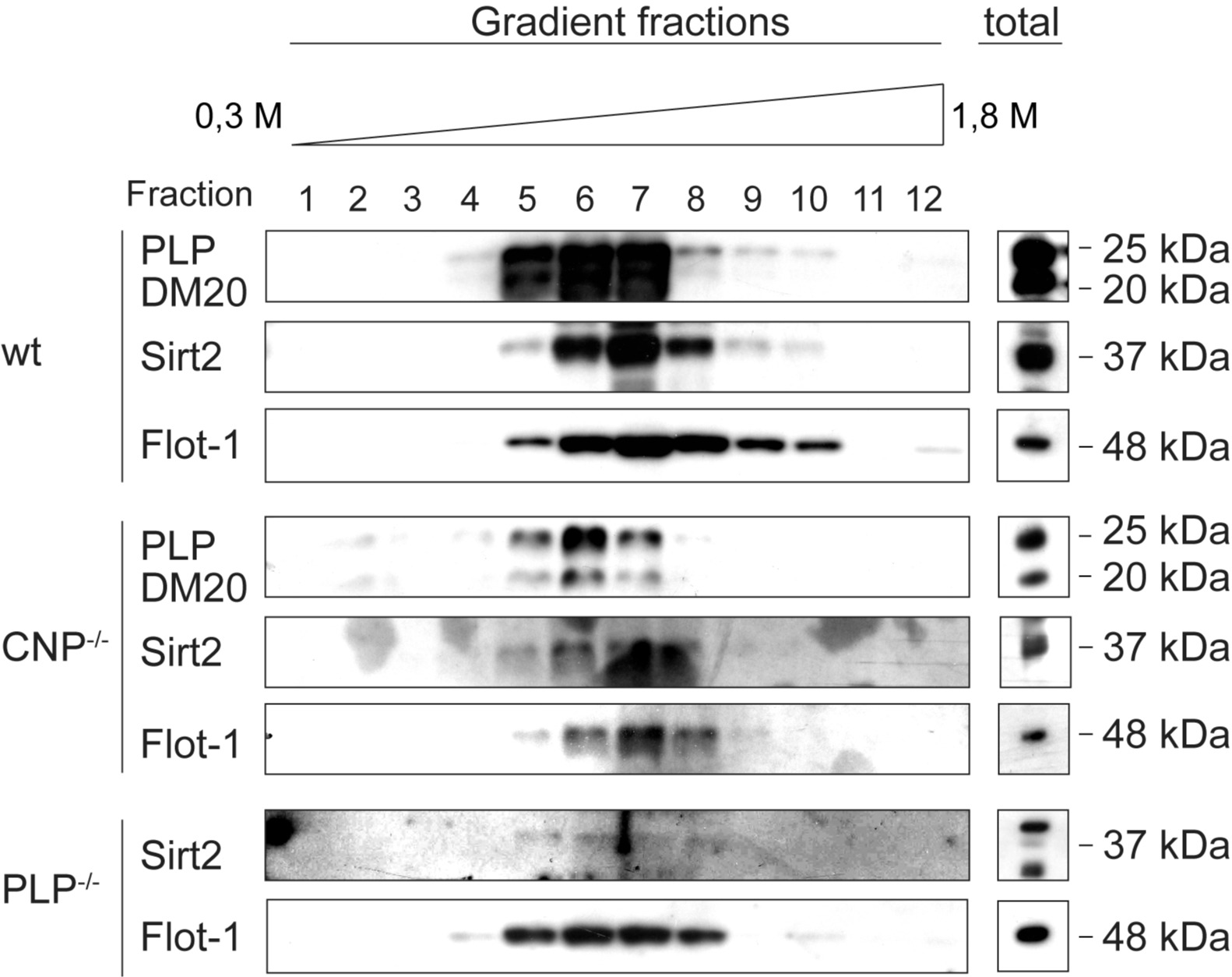

Supplement: S4 Fig — sEVs isolated from culture supernatants derived from equal numbers of wild-type, CNP-null, and PLP-null oligodendrocytes by differential ultracentrifugation followed by density gradient centrifugation (0.3–1.8 M sucrose gradient). Individual fractions were analyzed by Western blotting using established oligodendroglial sEV markers, PLP/DM20 (DM20 is a smaller isoform of PLP), Sirtuin-2, and Flotillin-1. Slight shifts in fractions between the markers indicate heterogeneity in sEV/exosome populations, which has been recognized previously [8]. Wild-type and mutant sEVs appear of similar density. Note that marker intensity appears weaker in mutant sEVs, reflecting a lower sEV yield from the same volume of starting material. Underlying images of blots can be found in S1 Images. (TIF) [file pbio.3000621.s006.tif]

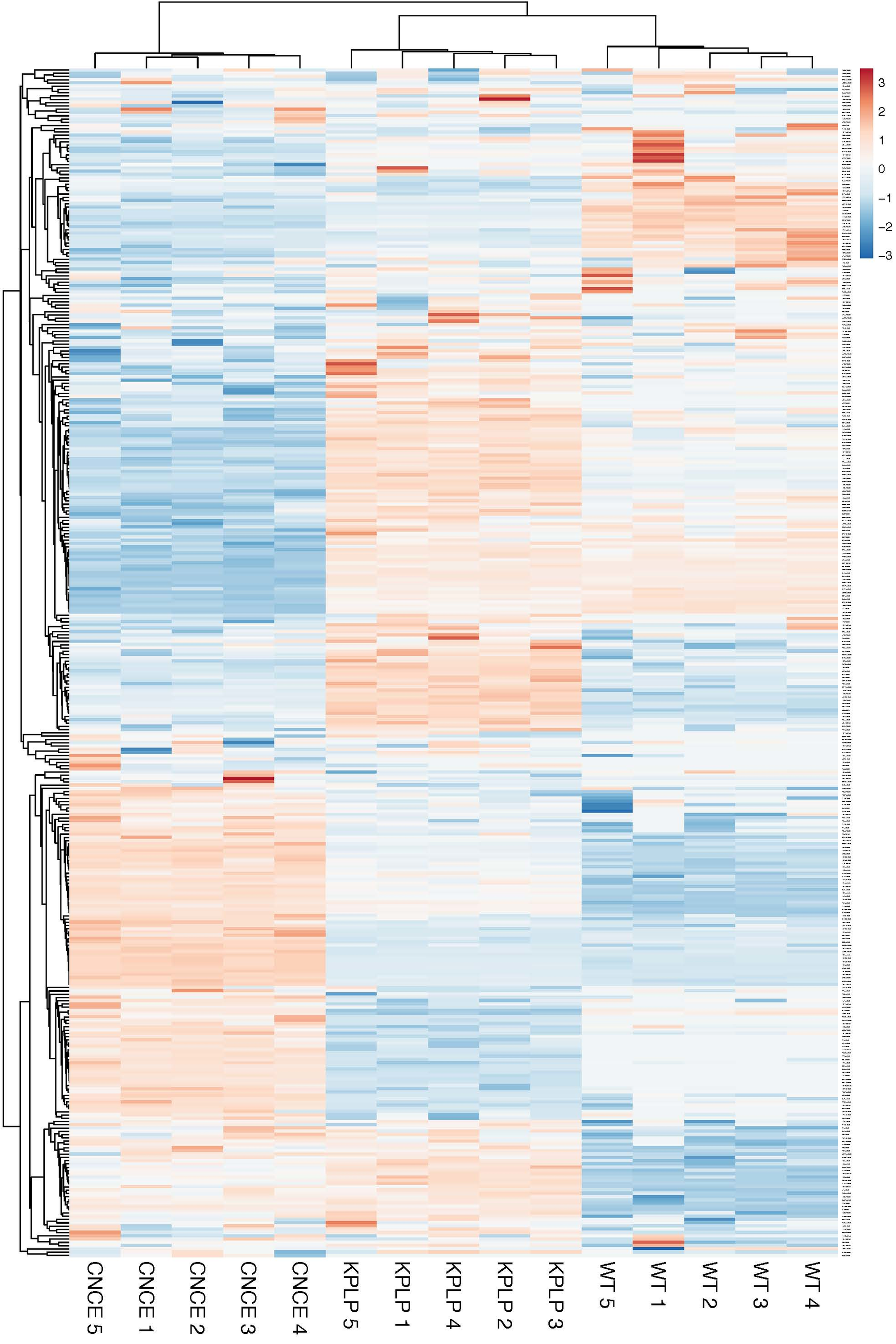

Supplement: S5 Fig — Relative expression values for all quantified proteins were used for an unsupervised hierarchical cluster analysis using the ClustVis Toolbox (PMID: 25969447). The analysis indicates clear separation of EVs from the different genetic backgrounds. Underlying data can be found in S2 Data. (TIF) [file pbio.3000621.s007.tif]

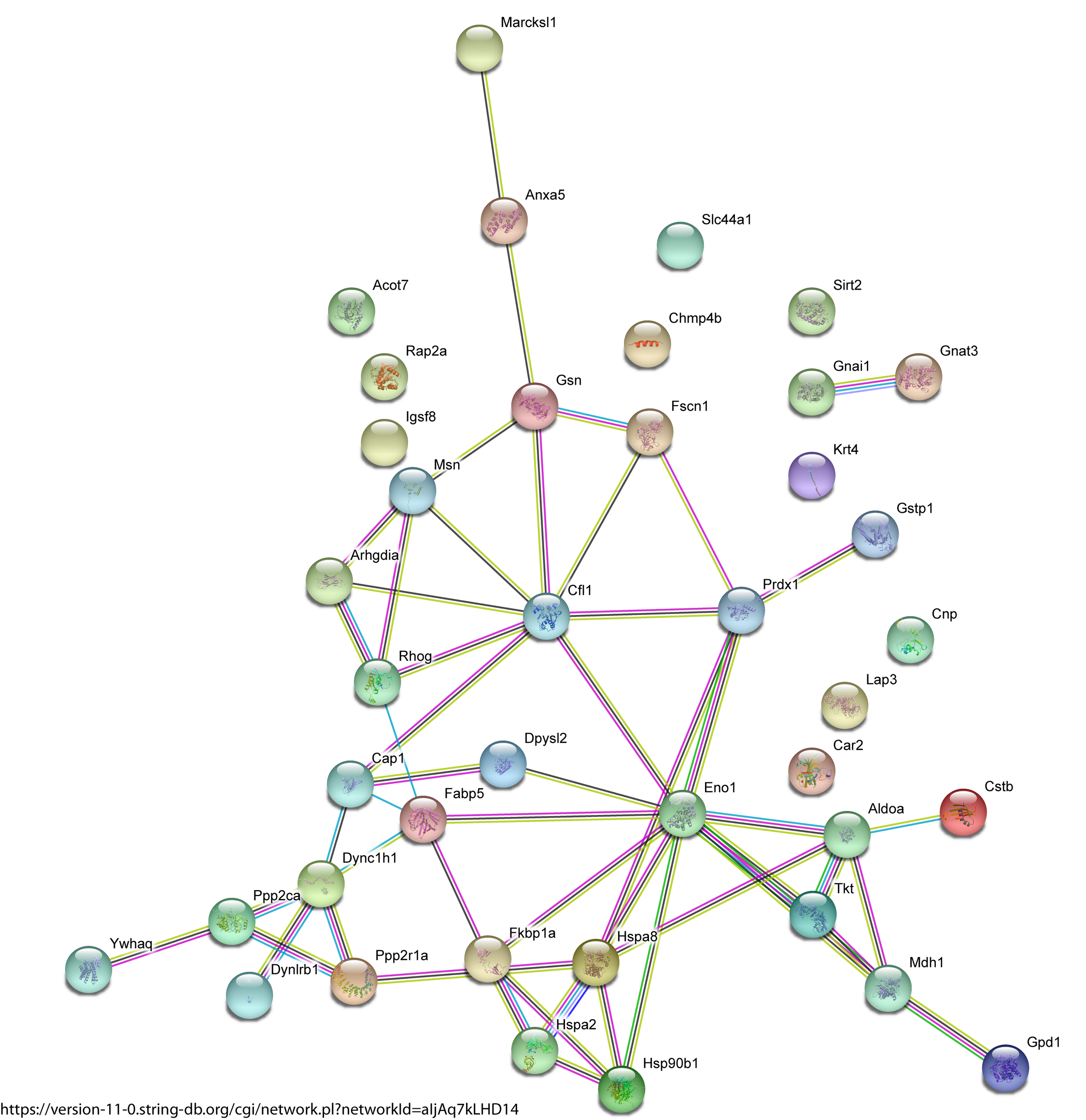

Supplement: S6 Fig — Details can be found at https://version-11-0.string-db.org/cgi/network.pl?networkId=aIjAq7kLHD14. (TIF) [file pbio.3000621.s008.tif]

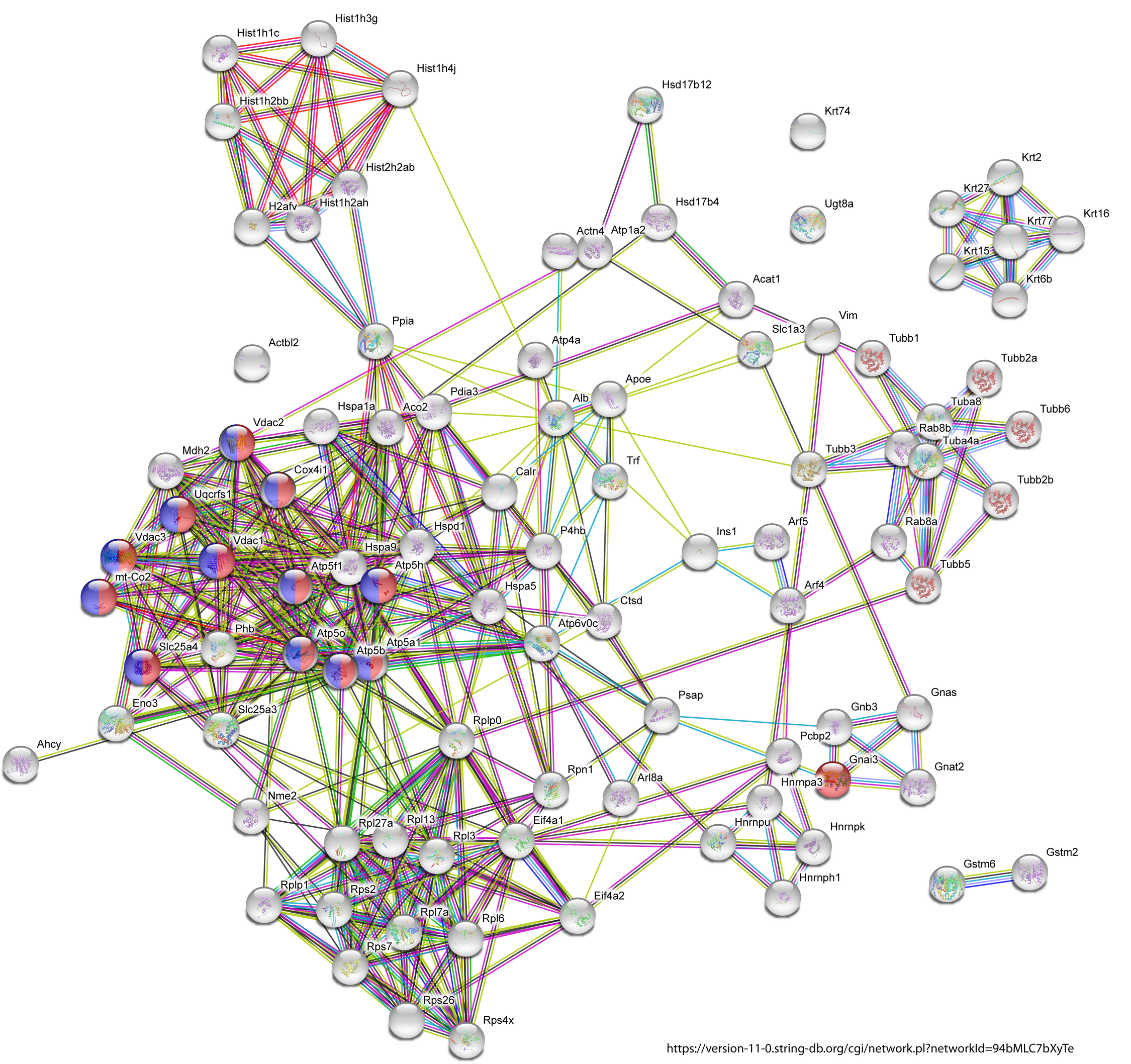

Supplement: S7 Fig — Details can be found at https://version-11-0.string-db.org/cgi/network.pl?networkId=94bMLC7bXyTe. (TIF) [file pbio.3000621.s009.tif]

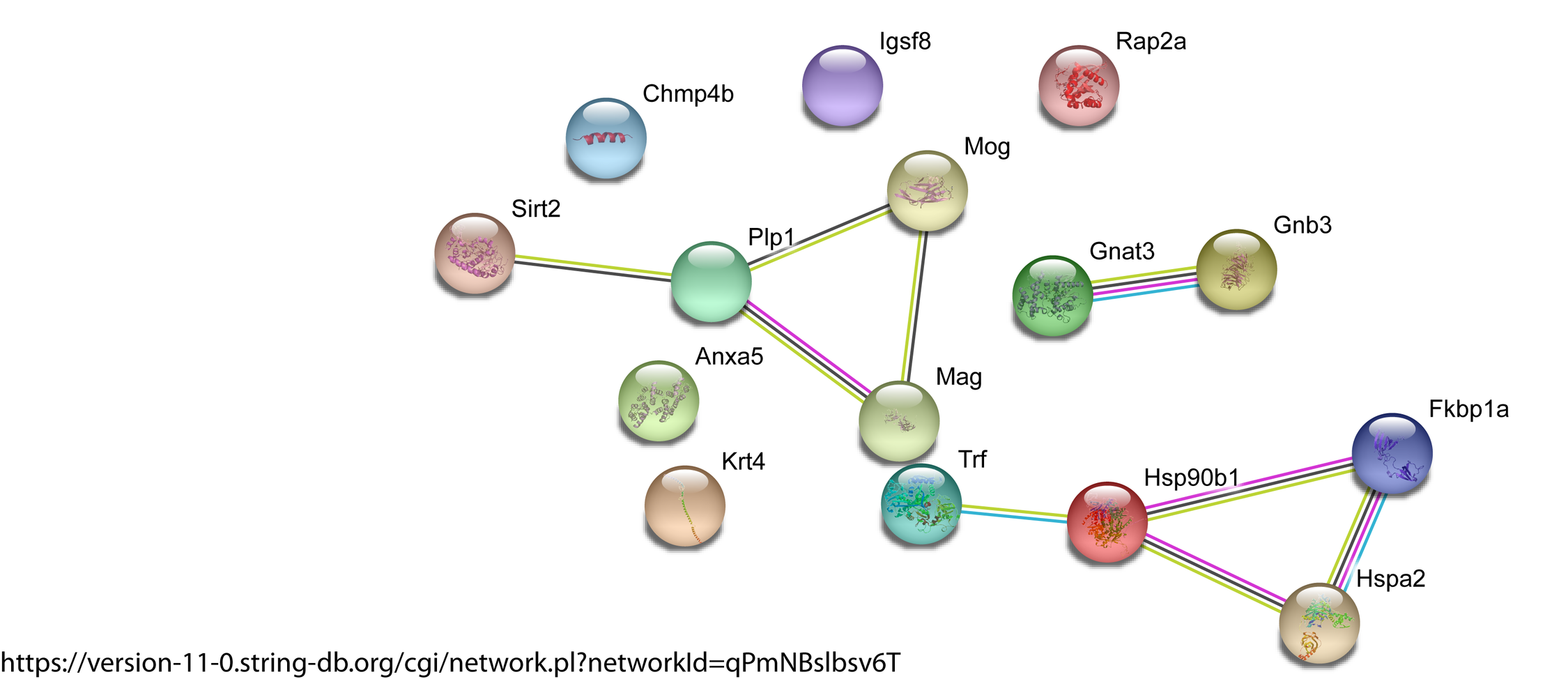

Supplement: S8 Fig — Details can be found at https://version-11-0.string-db.org/cgi/network.pl?networkId=qPmNBslbsv6T. (TIF) [file pbio.3000621.s010.tif]

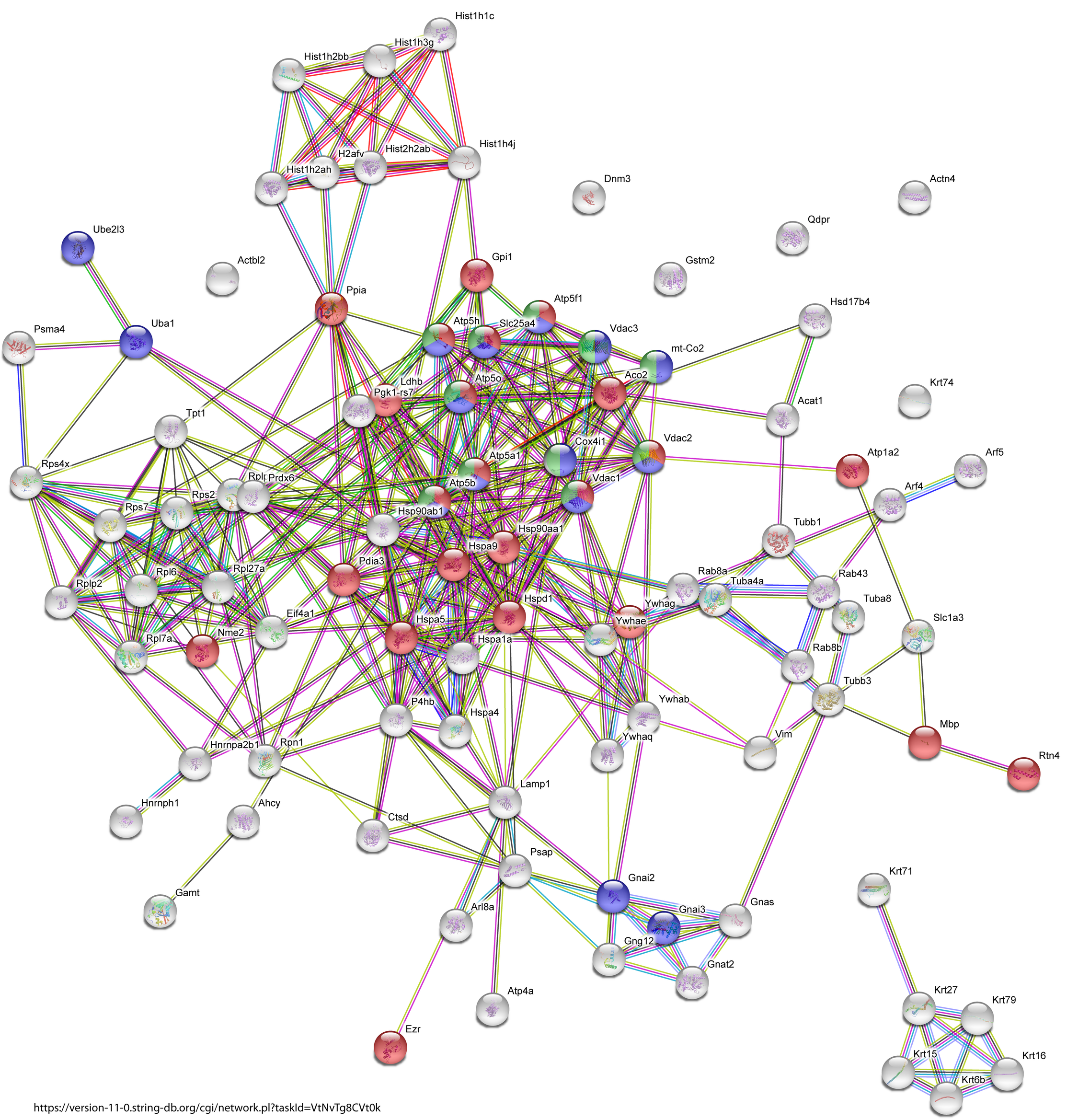

Supplement: S9 Fig — Details can be found at https://version-11-0.string-db.org/cgi/network.pl?networkId=jewb5zs2ixkl. (TIF) [file pbio.3000621.s011.tif]
