# Supplementary material for: Oligodendrocytes support axonal transport and maintenance via exosome secretion
Source: PLoS Biol. 2020 Dec 22;18(12):e3000621. doi: 10.1371/journal.pbio.3000621 (PMC7787684; doi:10.1371/journal.pbio.3000621)
Supplement: S1 Images — (PDF) [file pbio.3000621.s012.pdf]

Figure 1B

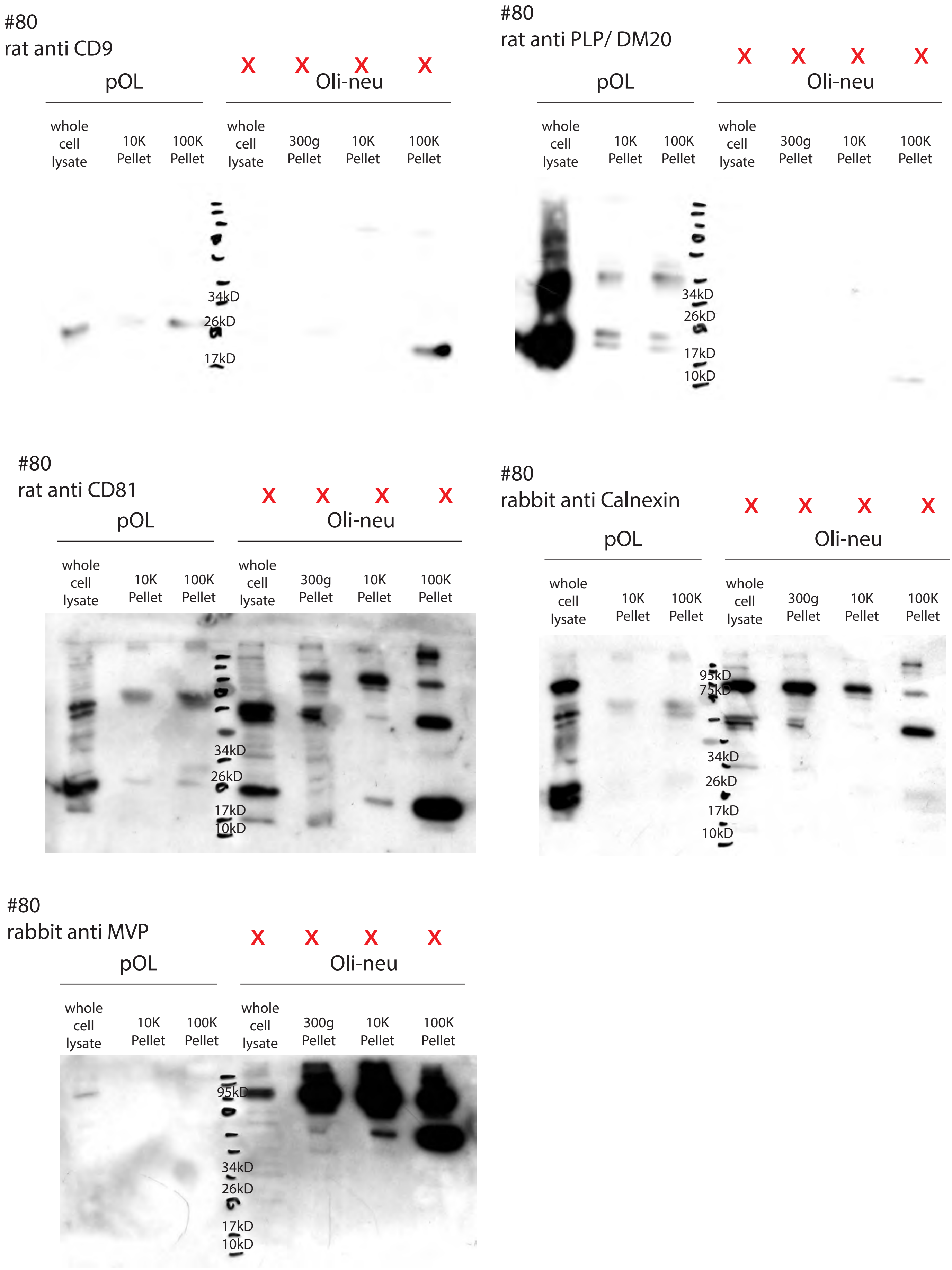

X = lanes not used in figure

Figure 1C

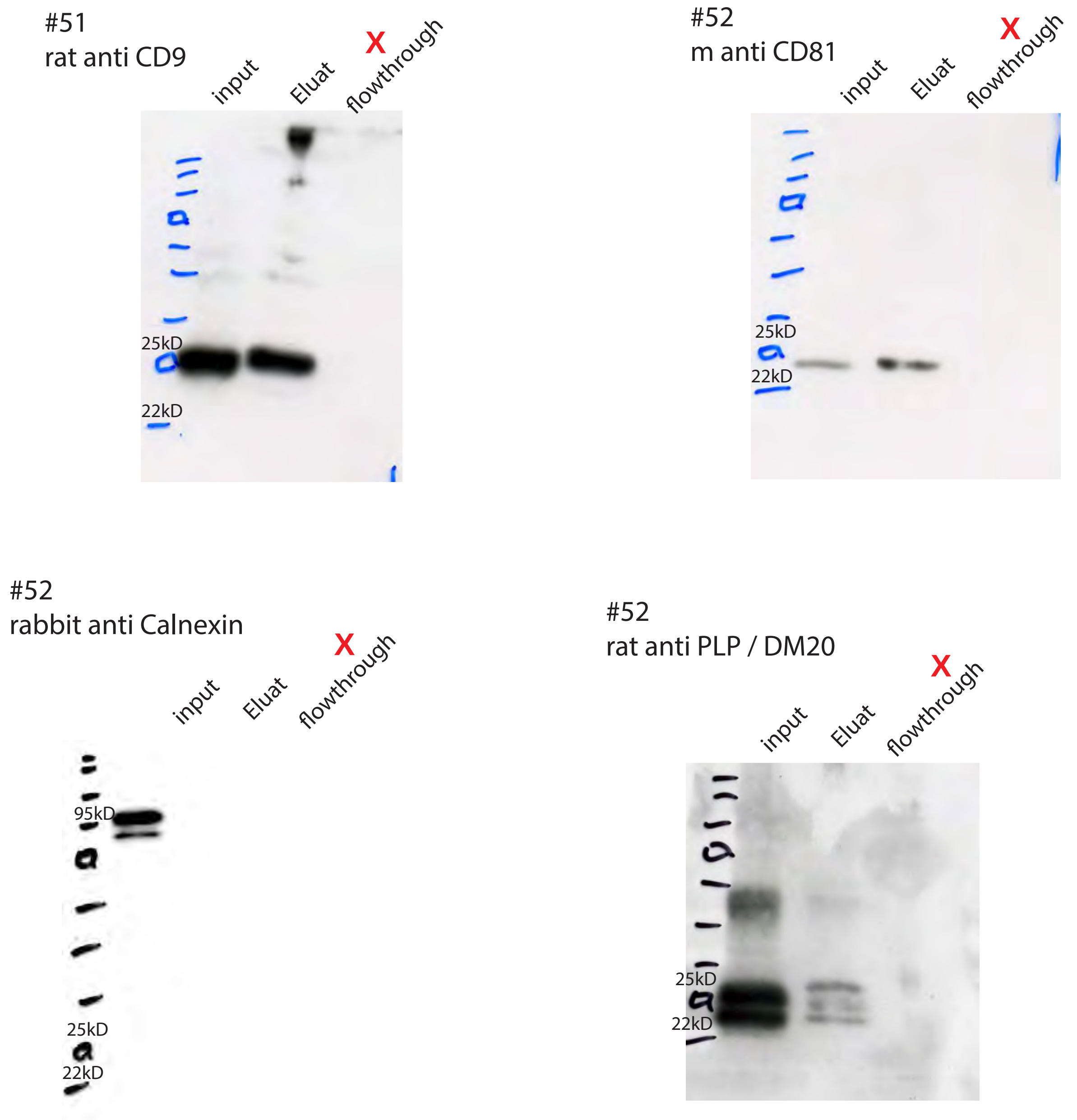

X = lane not used in figure

Figure 1D

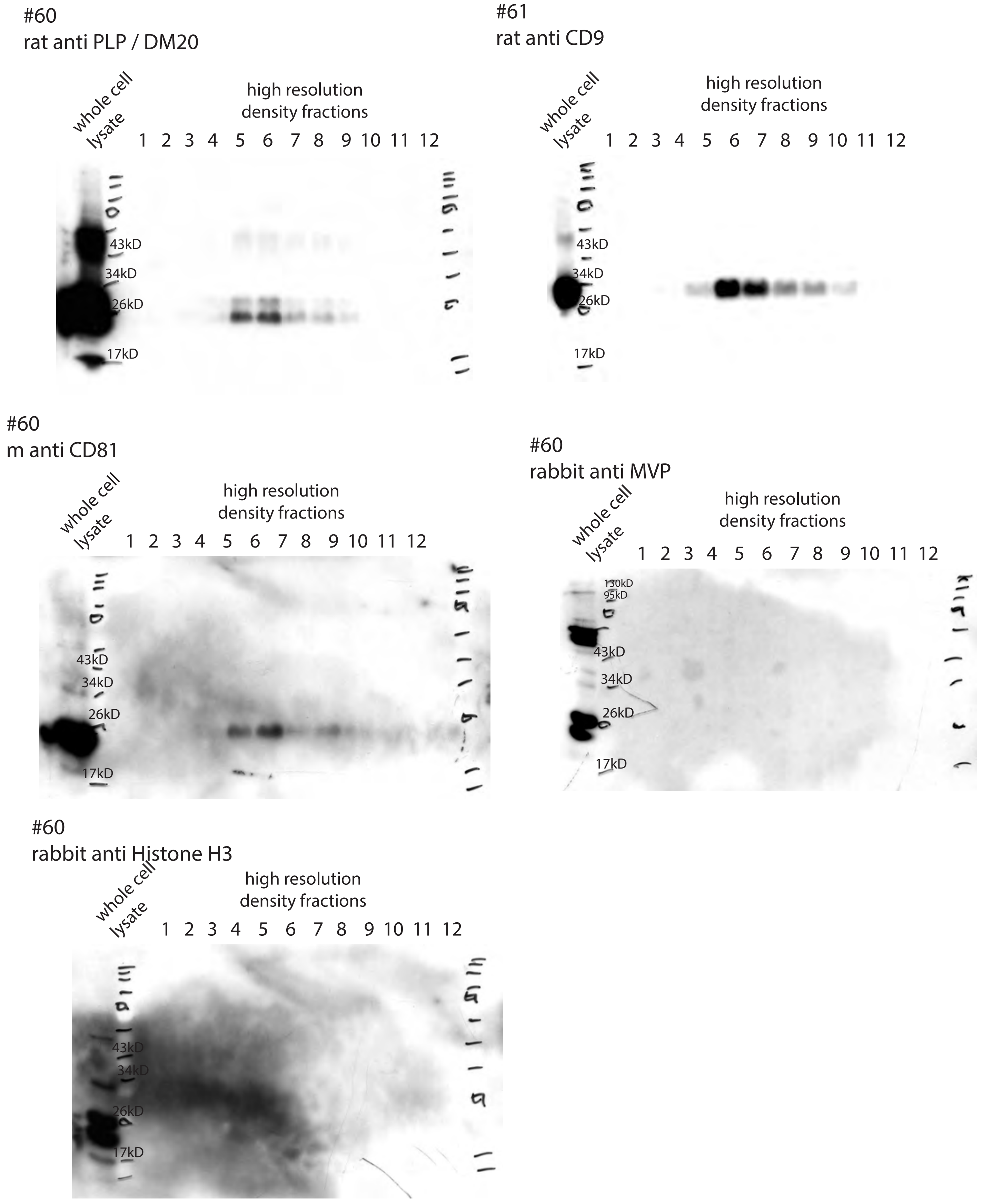
#60
rat anti PLP / DM20
whole cell lysate
high resolution density fractions
1 2 3 4 5 6 7 8 9 10 11 12
43kD
34kD
26kD
17kD
#61
rat anti CD9
whole cell lysate
high resolution density fractions
1 2 3 4 5 6 7 8 9 10 11 12
43kD
34kD
26kD
17kD
#60
m anti CD81
whole cell lysate
high resolution density fractions
1 2 3 4 5 6 7 8 9 10 11 12
43kD
34kD
26kD
17kD
#60
rabbit anti MVP
whole cell lysate
high resolution density fractions
1 2 3 4 5 6 7 8 9 10 11 12
130kD
95kD
43kD
34kD
26kD
17kD
#60
rabbit anti Histone H3
whole cell lysate
high resolution density fractions
1 2 3 4 5 6 7 8 9 10 11 12
43kD
34kD
26kD
17kD

Figure 1E

#62
rat anti PLP/ DM20

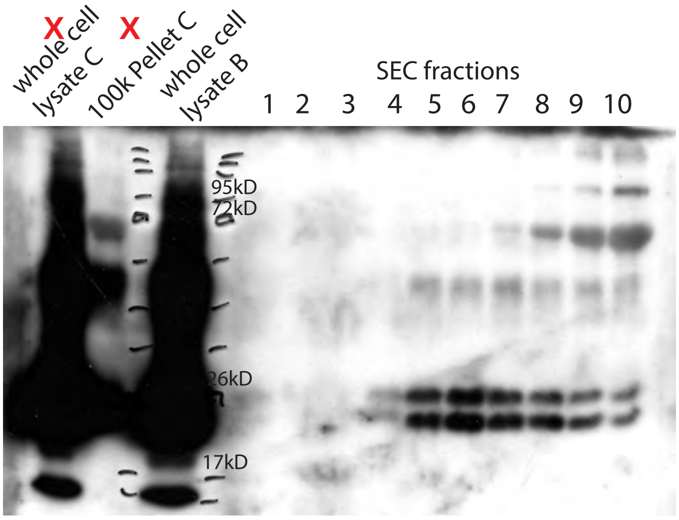

#63
rat anti CD9

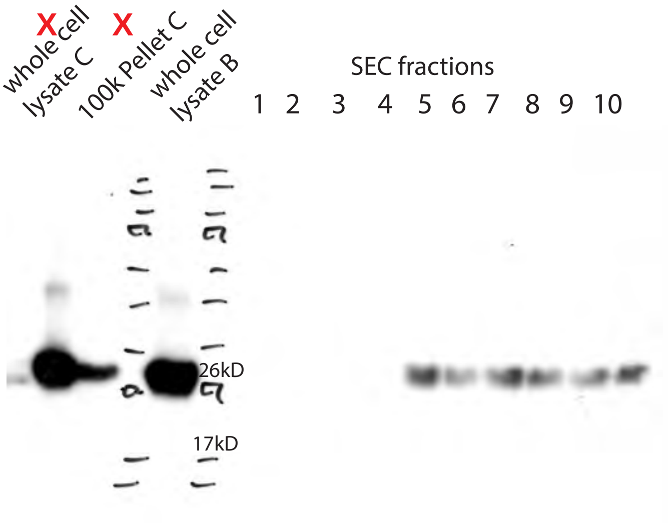

#62
rat anti Ago2

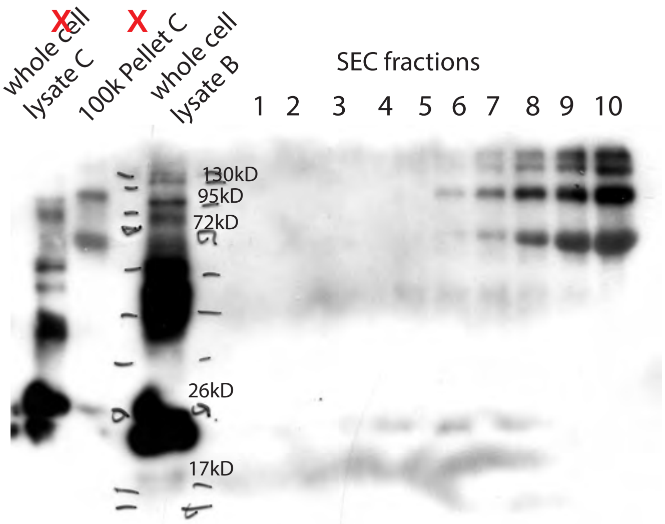

#62
mouse anti CD81

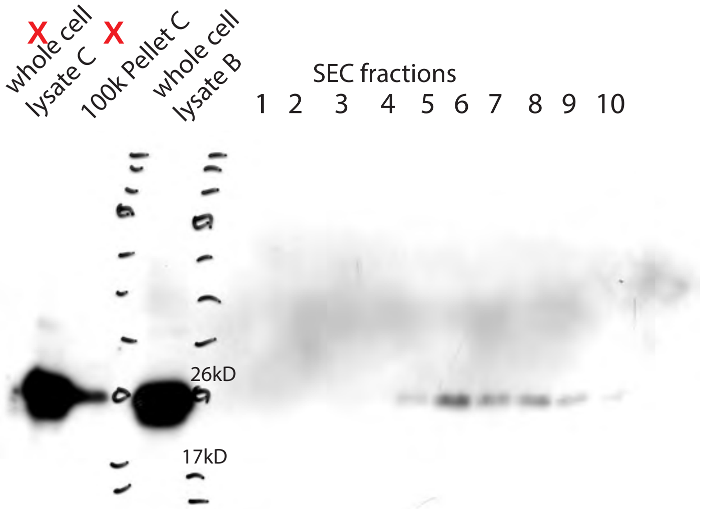

#62
rabbit anti MVP

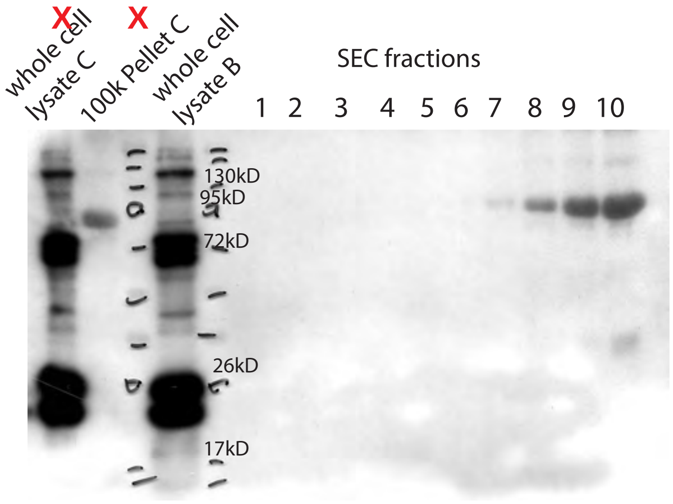

#62
rabbit anti H3

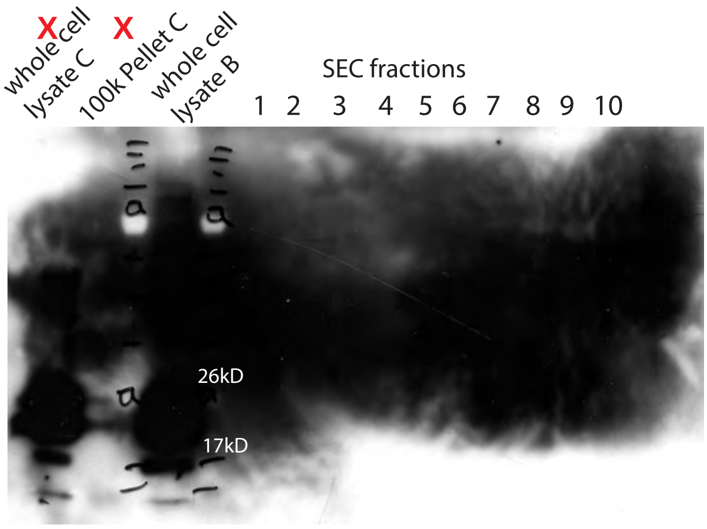

X = not used for Figure

Figure 3F

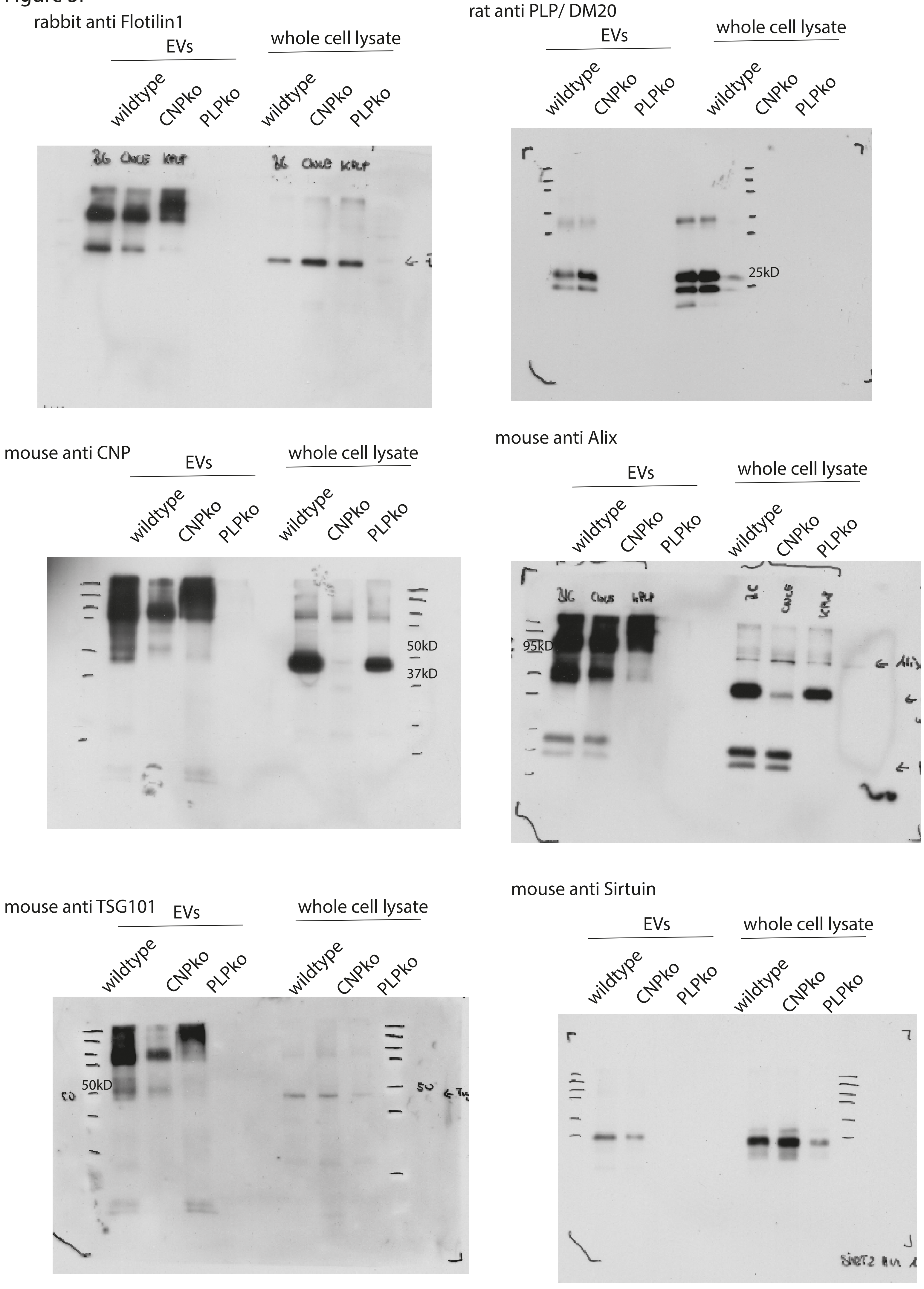
rabbit anti Flotilin1
EVs
whole cell lysate
wildtype
CNPko
PLPko
wildtype
CNPko
PLPko
rat anti PLP/ DM20
EVs
whole cell lysate
wildtype
CNPko
PLPko
wildtype
CNPko
PLPko
25kD
mouse anti CNP
EVs
whole cell lysate
wildtype
CNPko
PLPko
wildtype
CNPko
PLPko
50kD
37kD
mouse anti Alix
EVs
whole cell lysate
wildtype
CNPko
PLPko
wildtype
CNPko
PLPko
95kD
mouse anti TSG101
EVs
whole cell lysate
wildtype
CNPko
PLPko
wildtype
CNPko
PLPko
50kD
mouse anti Sirtuin
EVs
whole cell lysate
wildtype
CNPko
PLPko
wildtype
CNPko
PLPko

Figure 3F

rabbit anti HSP70

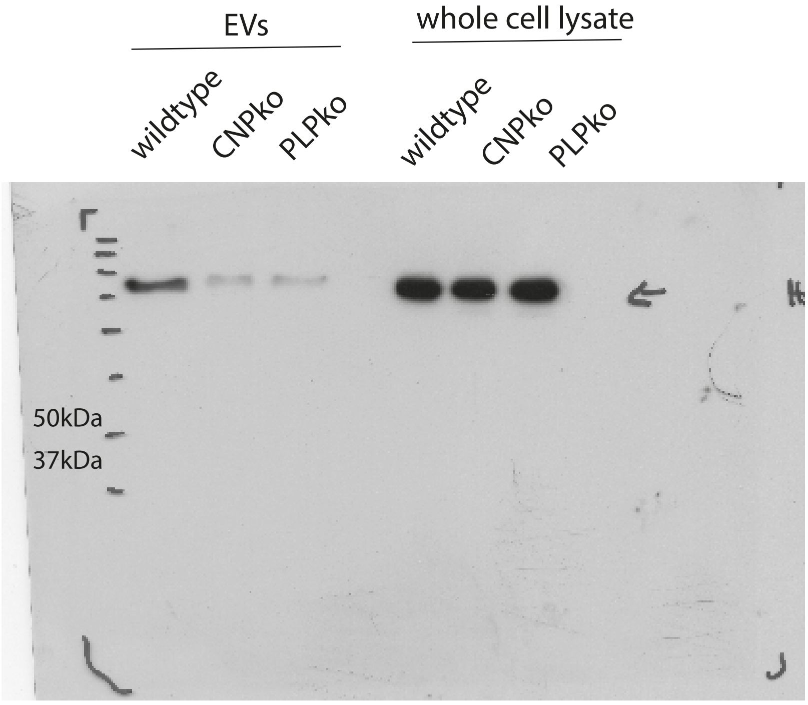

Figure 4E

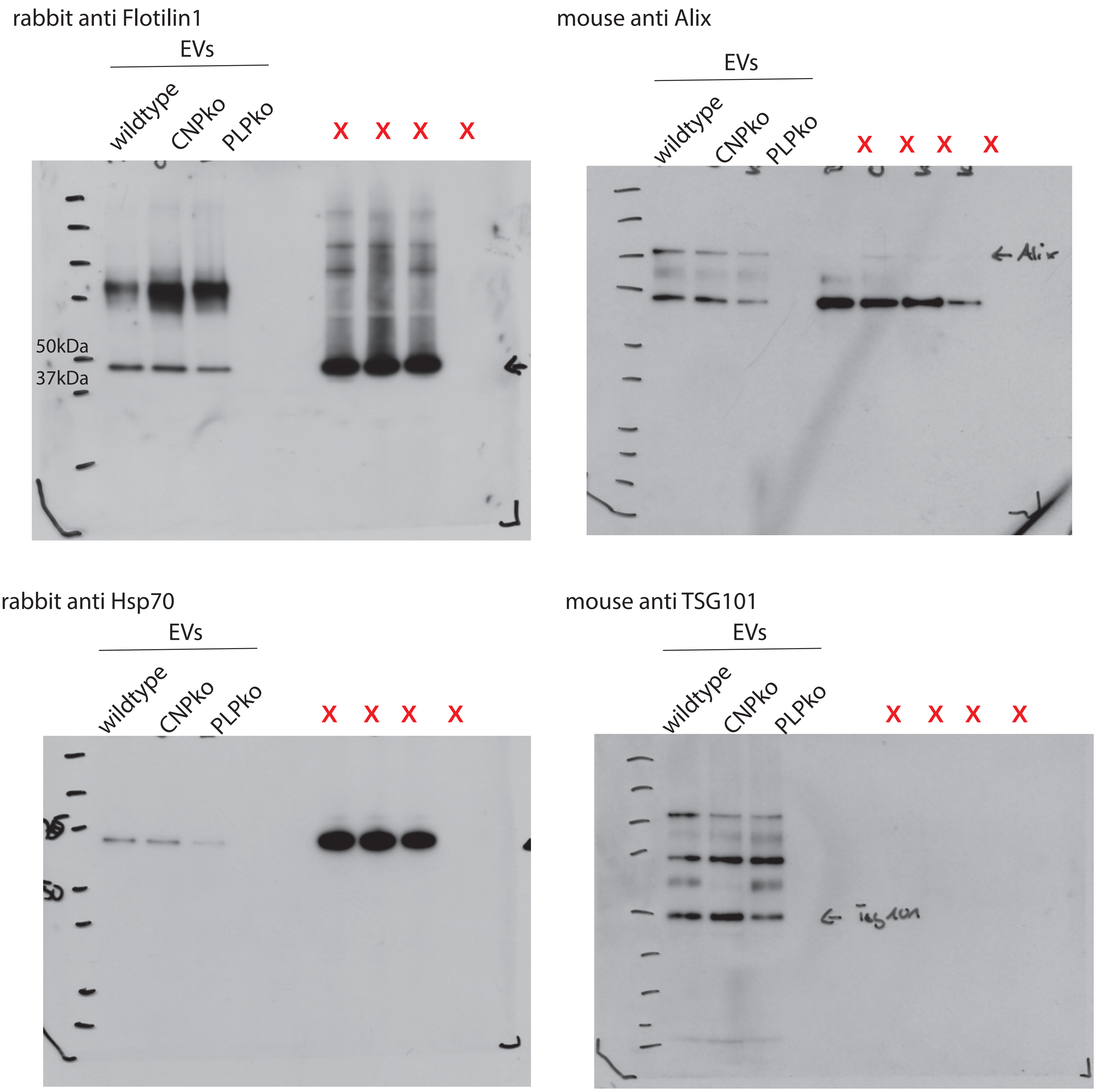

X = lanes not used in figure

Figure 4E

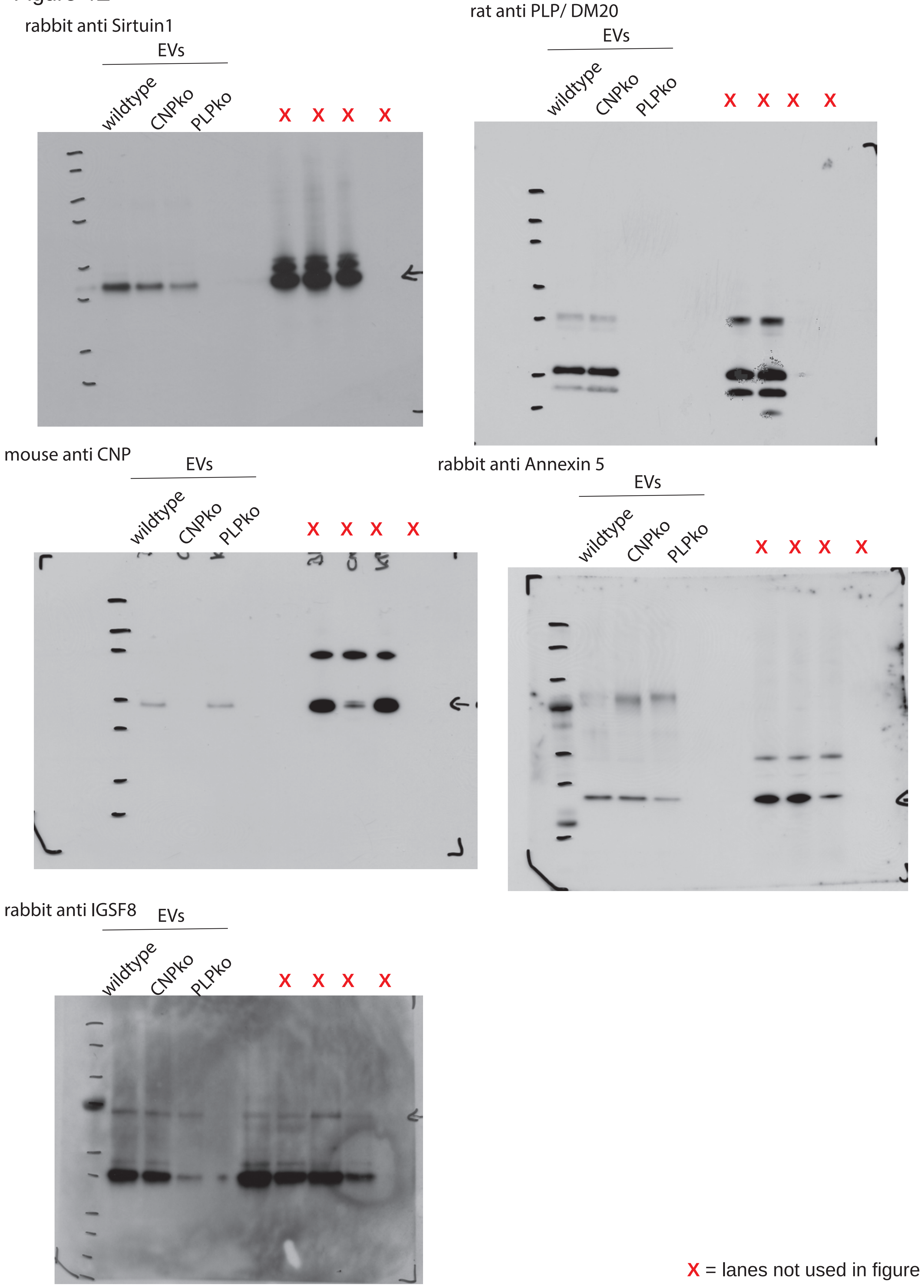
rabbit anti Sirtuin1
EVs
wildtype
CNPko
PLPko
X X X X
rat anti PLP/ DM20
EVs
wildtype
CNPko
PLPko
X X X X
mouse anti CNP
EVs
wildtype
CNPko
PLPko
X X X X
rabbit anti Annexin 5
EVs
wildtype
CNPko
PLPko
X X X X
rabbit anti IGSF8
EVs
wildtype
CNPko
PLPko
X X X X
X = lanes not used in figure

Figure 5A

rabbit anti hrGFP

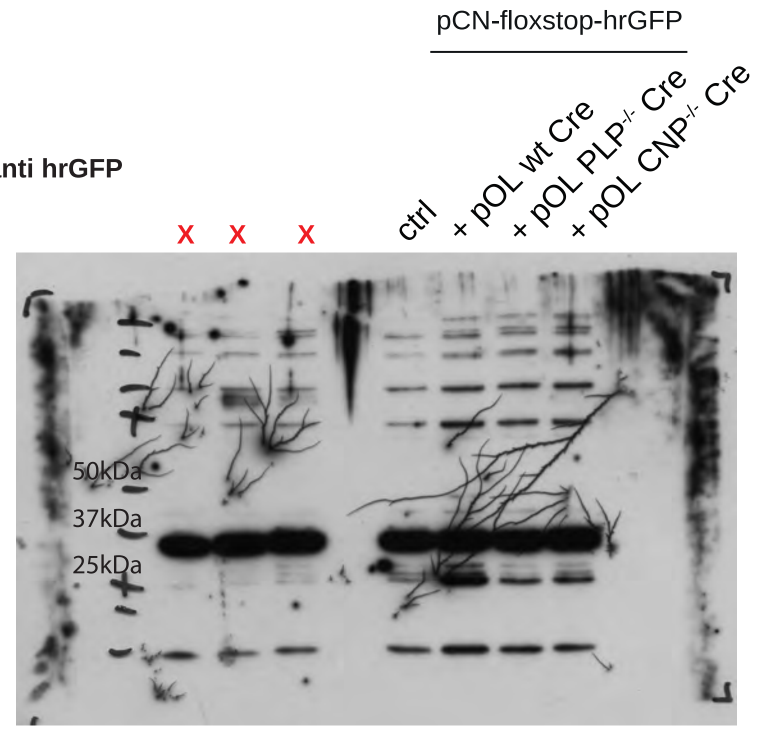

mouse anti Tubulin

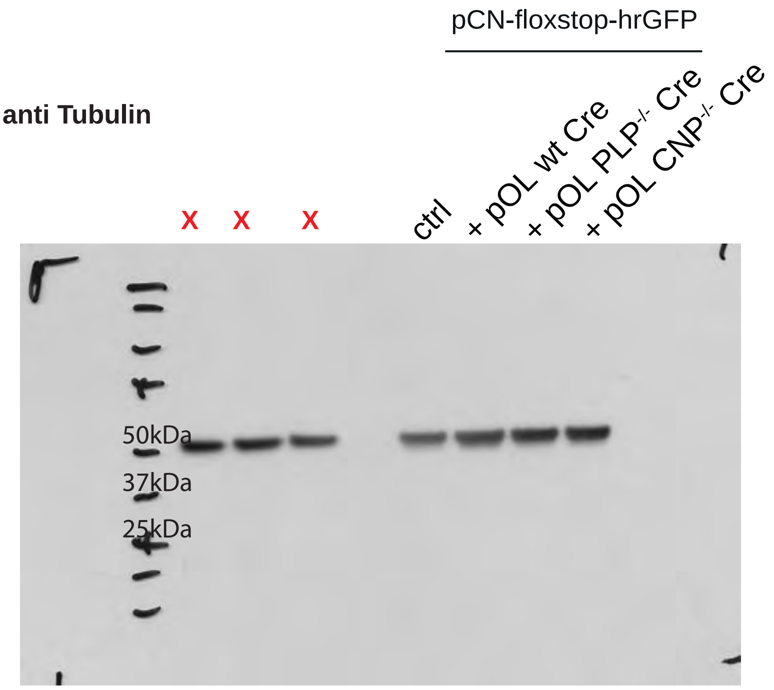

Figure S3B

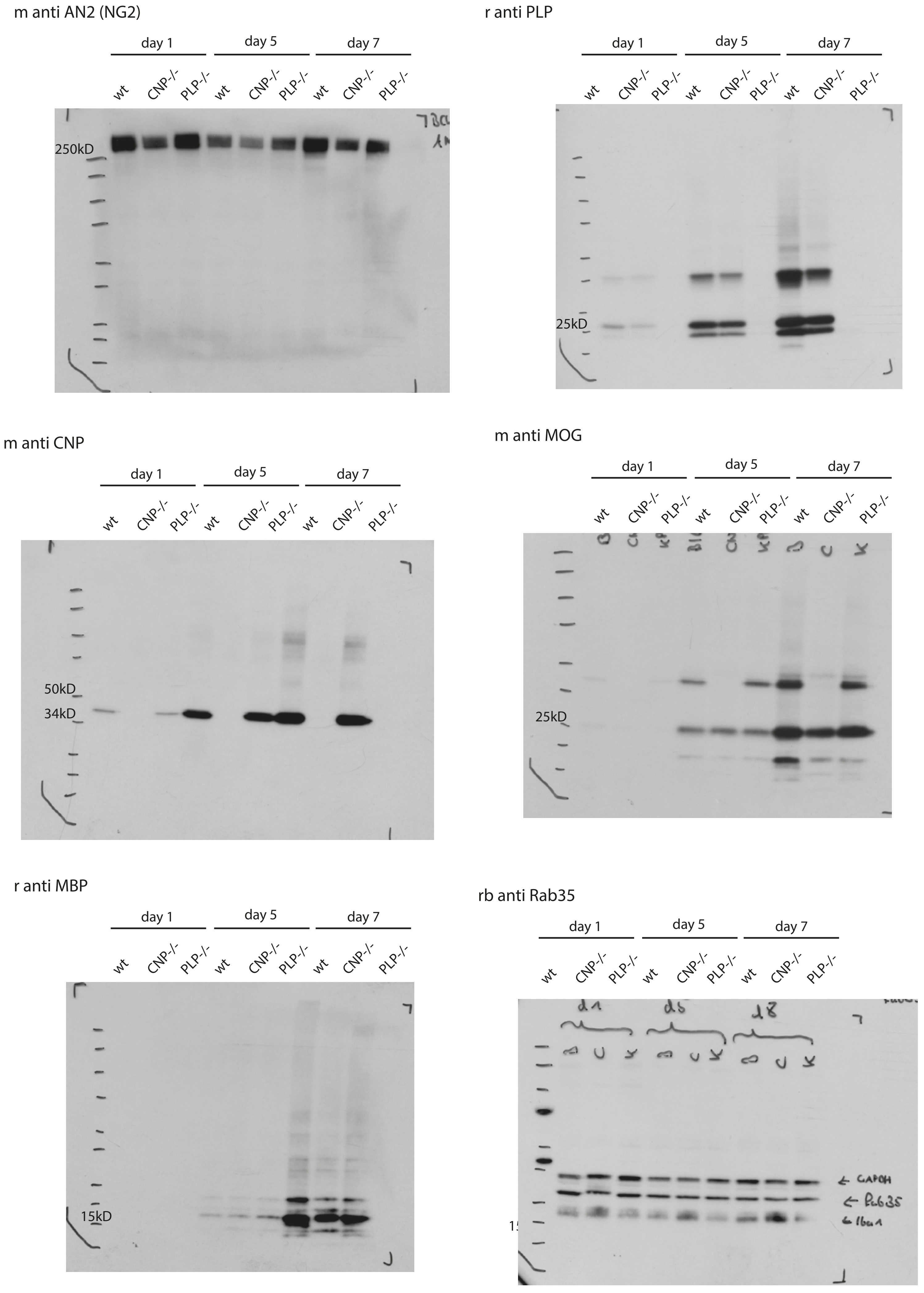
m anti AN2 (NG2)
day 1
day 5
day 7
wt
CNP-/-
PLP-/-
250kD
r anti PLP
25kD
m anti CNP
50kD
34kD
m anti MOG
25kD
r anti MBP
15kD
rb anti Rab35
← GAPDH
← Rab35
← Iba1

Figure S3B

rb anti GFAP

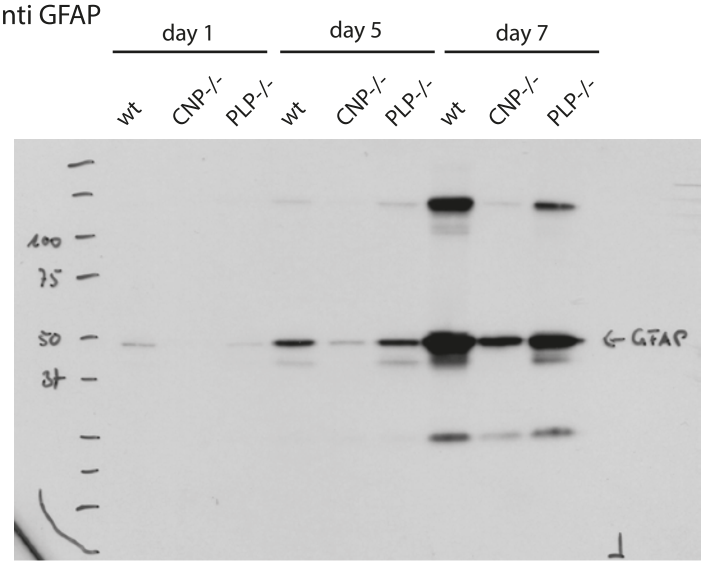

rb anti Iba1

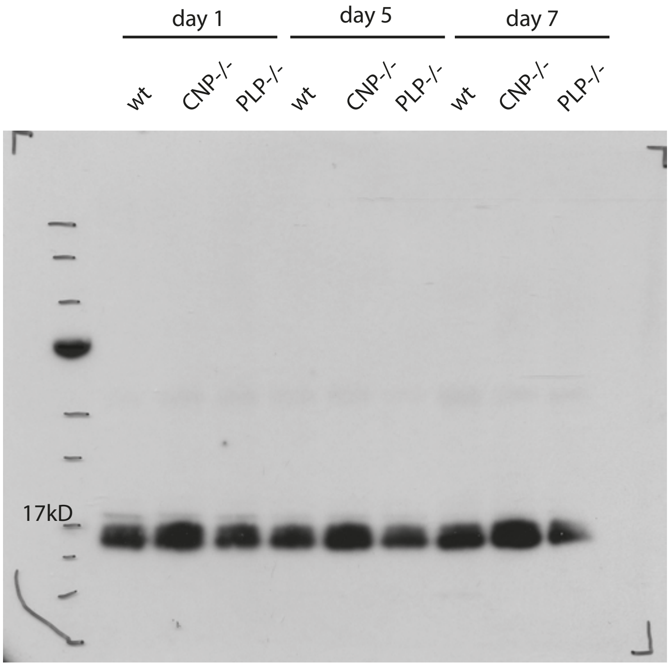

rb anti GAPDH

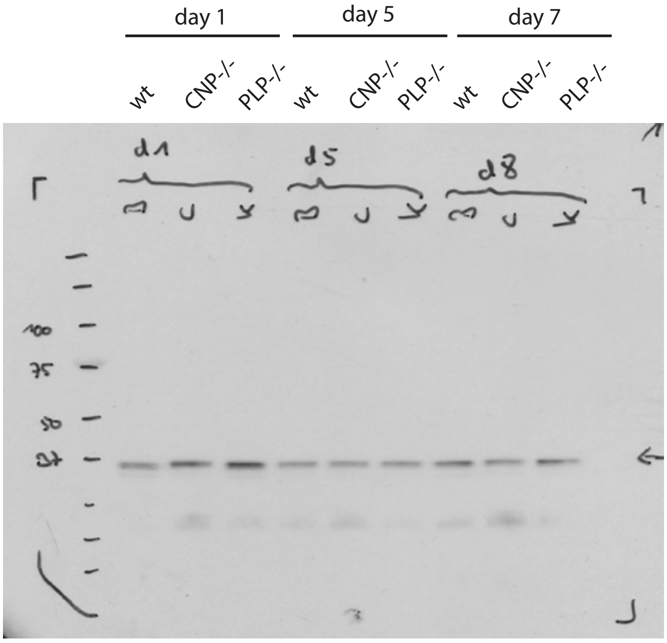

Figure S4

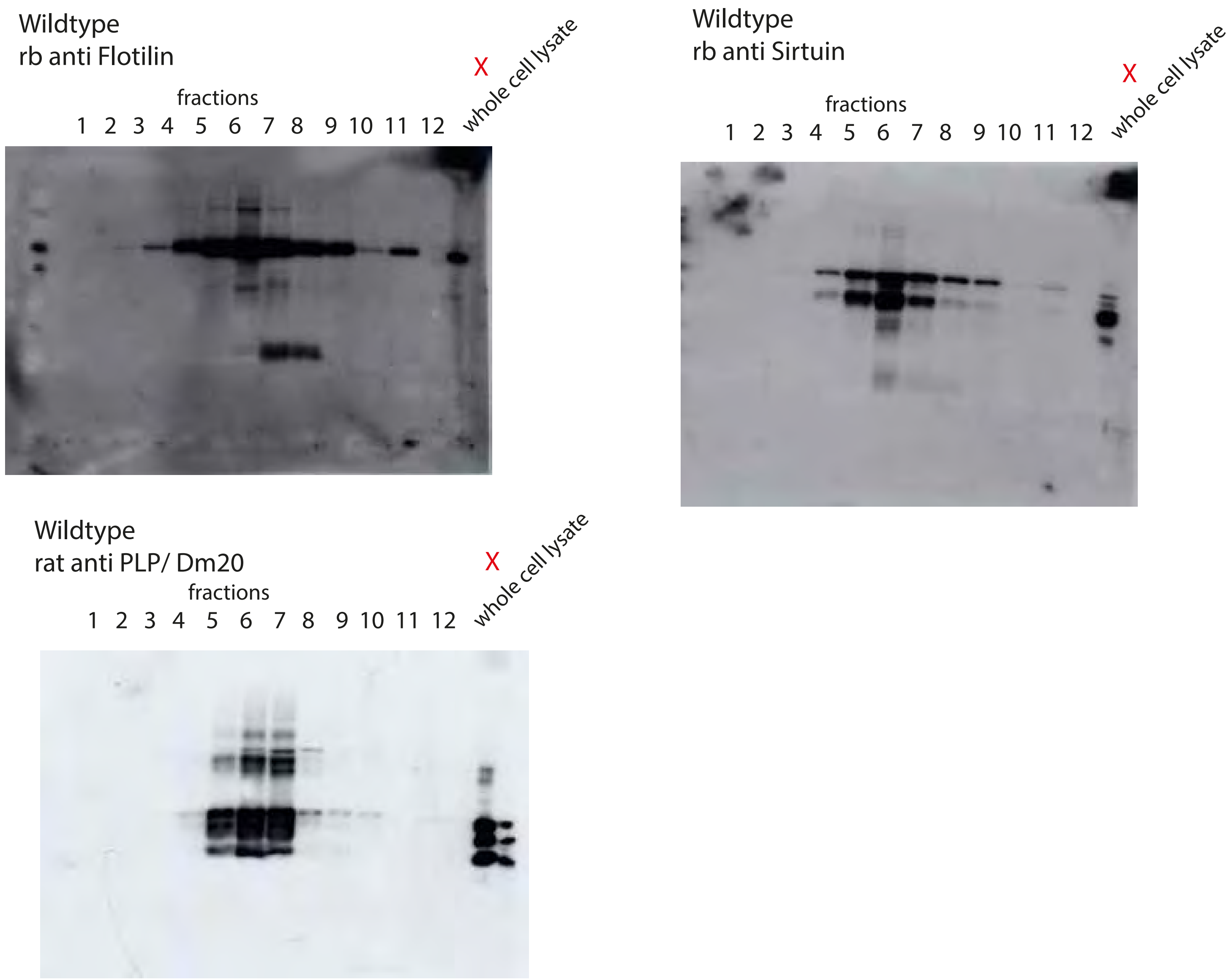
Wildtype
rb anti Flotilin
fractions
1 2 3 4 5 6 7 8 9 10 11 12
X
whole cell lysate
Wildtype
rb anti Sirtuin
fractions
1 2 3 4 5 6 7 8 9 10 11 12
X
whole cell lysate
Wildtype
rat anti PLP/ Dm20
fractions
1 2 3 4 5 6 7 8 9 10 11 12
X
whole cell lysate

Figure S4

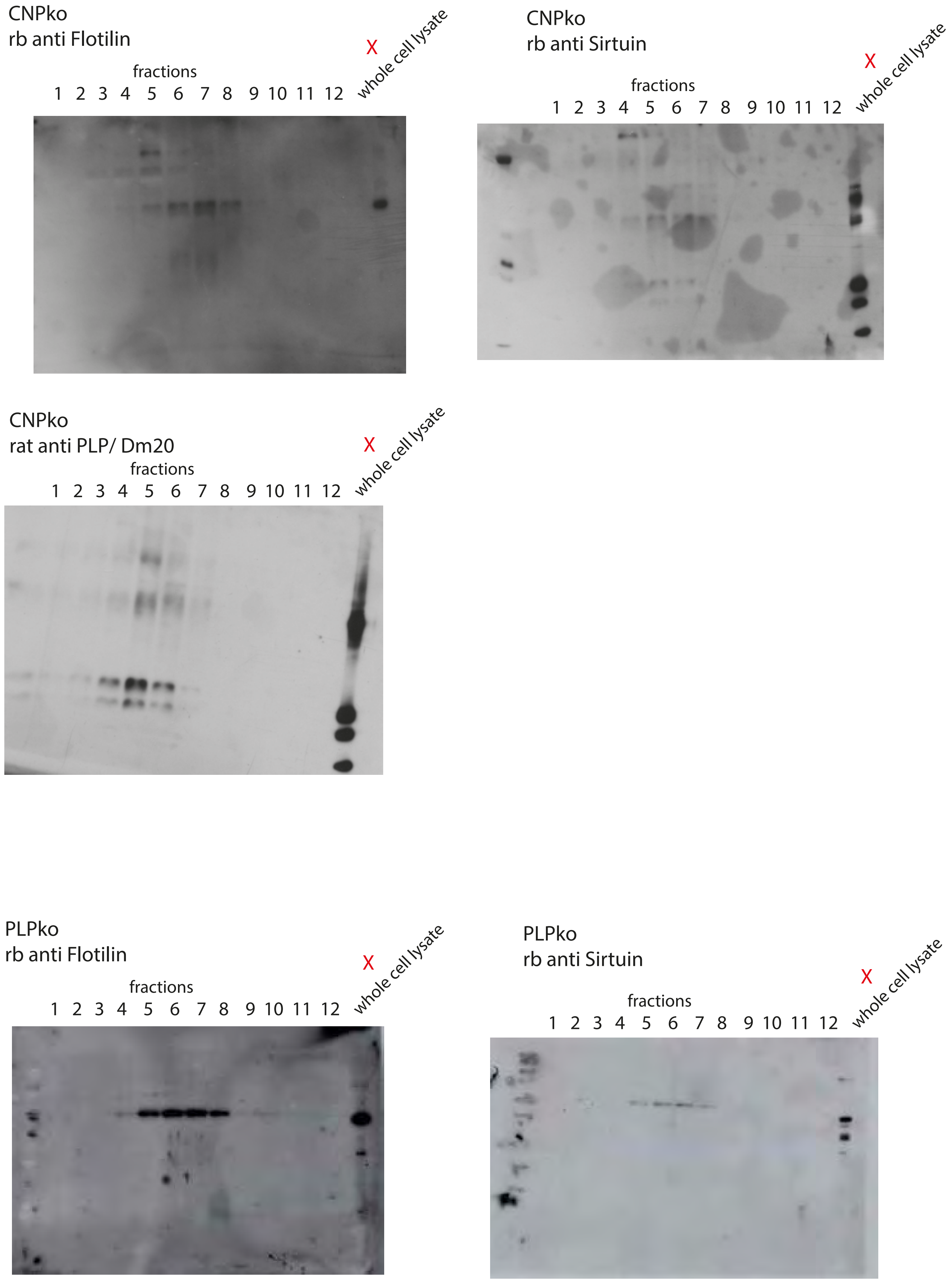
CNPko
rb anti Flotilin
fractions
1 2 3 4 5 6 7 8 9 10 11 12
X
whole cell lysate
CNPko
rb anti Sirtuin
fractions
1 2 3 4 5 6 7 8 9 10 11 12
X
whole cell lysate
CNPko
rat anti PLP/ Dm20
fractions
1 2 3 4 5 6 7 8 9 10 11 12
X
whole cell lysate
PLPko
rb anti Flotilin
fractions
1 2 3 4 5 6 7 8 9 10 11 12
X
whole cell lysate
PLPko
rb anti Sirtuin
fractions
1 2 3 4 5 6 7 8 9 10 11 12
X
whole cell lysate
