# Supplementary material for: Oligodendrocytes support axonal transport and maintenance via exosome secretion
Source: PLoS Biol. 2020 Dec 22;18(12):e3000621. doi: 10.1371/journal.pbio.3000621 (PMC7787684; doi:10.1371/journal.pbio.3000621)
Supplement: Attachment — Submitted filename: critique-plos biology.docx [file pbio.3000621.s013.docx]

The manuscript “Oligodendrocyte-derived exosomes promote axonal transport and axonal long term Maintenance” by Frühbeis et al described an interesting observation that oligodendroglial exosomes support neurons by promoting fast axonal transport, especially under conditions of oxidative stress and nutrient deprivation. The authors also explored the quantitative and cargo changes of mutant oligodendroglia-derived exosomes which are unable to support neurons. The study unveil a potentially interesting and new mechanism how oligodendroglia support axonal health. However, certain experiments are incomplete; figures are confusing with unclear legend, missing of important controls, as well as several major flaws significantly undermine the conclusion, as summarized below:

1. Figures are difficult to understand and the legend is very incomplete.

2. Cre packaging can be altered in mutant oligodendrocytes.

3. There is no ND condition alone, the treatment paradigm is incomplete. it is possible that oligodendrocyte-derived supernatant also contains toxic factors

4. axonal transport vs. overall neuronal health
