# Supplementary material for: Oligodendrocytes support axonal transport and maintenance via exosome secretion
Source: PLoS Biol. 2020 Dec 22;18(12):e3000621. doi: 10.1371/journal.pbio.3000621 (PMC7787684; doi:10.1371/journal.pbio.3000621)
Supplement: Attachment — Submitted filename: Reviewers response_final 2020-07-23.docx [file pbio.3000621.s014.docx]

**PLOS Biology Resubmission (PBIOLOGY-D-19-03575R1)**

**Letter in response to reviewer’s comments**

(reviewers’ comments in black, authors’ response in red)

We thank all reviewers for the quite positive evaluatio, the acknowledgement of our work and the constructive input. We planned to comprehensively address all individual comments and in particular to perform additional axonal transport experiments. These are sophisticated experiments, all performed with limited primary cell material and sEVs, isolated in elaborate procedures and requiring coordination of several experimental steps and setups. Functional experiments, which always have to include the appropriate controls, require an enormous amount of freshly isolated sEVs, which are limited in amount when harvested from postmitotic primary oligodendrocytes. However, due to the COVID pandemics and university lockdown, we unfortunately were not able to perform these laborious axonal transport experiments in the past months and think that, at this point, it is more productive to elaborate on the existing solid body of data. We thus focused the manuscript on the nutrient deprivation experiments and the comparison between wildtype and mutant oligodendrocyte-derived sEVs, to deliver the main message more clearly and to avoid confusion. Please be aware, that the relevant control is the direct comparison of EV-treatment versus EV-depletion highlighting the impact of sEVs under this condition. We provide additional data regarding sEV characterization to demonstrate that we put strong emphasis in working with best-defined and physiologically representative sEV-fractions (an important prerequisite for reproducible functional studies on EVs). We hope for your understanding and agreement that this work is of solid quality and provides a valuable contribution to the scientific community.

Short overview over main manuscript changes:

- The title was changed to reflect more the focus on the functional failure of mutant-derived exosomes (as suggested by Reviewer 3)
- Figures 1 and 2 were moved to Supplementary Material to avoid confusion with distinct experimental protocols revealing the axonal transport assay as coherent set of experiments. These figures depict pilot experiments (built on experiments performed in our previous publications). We think the data are valid and provide important additional information and therefore show them as extra data.
- We included an additional Figure 1 showing basic characterization of oligodendrocyte-derived sEVs to convince the reader that the sEV fraction applied in the functional assays is a well-defined exosome-like fraction.
- We intensively discussed the points raised by all reviewers

**Reviewer 1:** Graca Raposo – note that this reviewer has waived anonymity.
In this manuscript Frühbeis and collaborators investigate how the intricate intercellular communication between glial cells and neurons can ensure long-term integrity of neuronal axons. In particular they investigate in detail how small Extracellular Vesicles with features of exosomes released by oligodendrocytes can support neurons and axonal transport even in stress conditions (oxidative stress, nutrient deprivation).

The team has previously reported how communication within the CNS can largely rely on a loop on intercellular communication based on release and transfer of exosomes in between glial cells and neurons. The present study extends these initial observations and brings light on how EV scan also support axonal integrity.

In my opinion the manuscript is very complete and detailed with a lot of appropriate controls. The results support the conclusions and certainly sets novel concepts in the understanding of how such loop of intercellular communication is also important for axonal maintenance and have implications for molecular basis of neurodegenerative diseases.

In the moving field of Extracellular Vesicles, the authors are very cautious with the nomenclature and it is very clear for non-advised readers in the field.

I have only 2 minor points:
The authors have previous characterized the EVs secreted by oligodendrocytes, although it would be may useful in the manuscript to include a small supplement with the minimal characterization of the vesicles?

Thank you very much for the appreciation of our work. We actually agree that information on the characteristics of the sEV-fractions are important for this functional study and the reader is not completely referred to our previous manuscripts to retrieve that data. We therefore included a new Figure 1, regarding EV-characterization and largely provide data (markers, isolation procedures) that cannot be found in these previous manuscripts. Please also be aware that there is a considerable body of information on oligodendroglial (OL) sEV characteristics also contained in Figures 3+4 (including proteomics) comparing wild-type and mutant OL-derived sEVs.

The authors show, using different methods a reduction, both quantitative and qualitative, of sEVs derived from CNP and PLP-null oligodendrocytes. They focused then on downregulated candidates though it is not very clear why the levels of TSP CD63/CD81 are not altered if the numbers of sEVs are reduced?

In quantitative proteomics, the presented data were normalized to the total amount of peptides detected in the sample in order to reflect the relative content of an individual EV-protein between the samples (Supplementary Table 1, expressed in parts per million-ppm). Hence the reduced release rates are wiped out and the fact that CD63 and CD81 are stable between wt, PLP- and CNP-null EV-samples actually confirms normalization (see page 9, top). Hence, the data reveal those proteins that are downregulated per se in the sEVs. Since the TSPANs CD63 and CD81 are often correlated with specific subpopulations, it seems that these subpopulations are not affected by the mutations.

Are we dealing with different MVB subpopulations that are affected in the null PLP and null CNP?
I am unsure whether this point will be clear for all readers.

As also mentioned above, we do not have evidence that specific subpopulations of EVs or MVBs are affected. MVB ultrastructure and overall morphology is similar in wt and mutant OL (comment added page 7), rather the mislocalization of the MVBs seems relevant for the reduction of sEV numbers. Which factors are responsible for the qualitative differences of mutant sEVs and whether endosomal protein sorting to MVB ILVs is affected requires further studies. See discussion page 14.

**Reviewer 2:**
The manuscript "Oligodendrocyte-derived exosomes promote axonal transport and axonal long term Maintenance" by Frühbeis et al described an interesting observation that oligodendroglial exosomes support neurons by promoting fast axonal transport, especially under conditions of oxidative stress and nutrient deprivation, which represents a potentially interesting and new mechanism how oligodendroglia support axonal health. The authors also explored the quantitative and cargo changes of mutant oligodendroglia-derived exosomes and concluded that these changes in exosomes is one of the possible mechanisms underlying glia-dependent secondary axonal degeneration in these mutant mice. However, this study, at its current format, suffers several major weaknesses, including design flaws, incompletion in certain experiments; confusing organization of several figures with unclear legend, and missing of important controls, which significantly undermine the conclusion.

Major comments:
1. The conclusion that oligodendrocytes-derived sEV promotes axonal transport is not convincing in Figure. 1. Most of the results presented in this figure is not different. The significance of pausing time on the axonal transport and health is not clearly described. In addition, different fractions such as 10K or 100K supernatant was used in the treatment in other Figures. These fractions were not used in this experiment, making it unclear how the baseline effect of these fractions is. In addition, the important control, the nutrient deprivation (ND) alone is lacking, making it difficult to assess the effect of different fractions on these neurons. Moreover, the use of ND in the manuscript is misleading, as it refers to the treatment of 100K supernatant from oligodendrocyte conditioned medium, but not the nutrient deprivation alone. It is important to recognize that there are other factors in oligodendrocyte conditioned medium that promotes neuronal survival, such
as Wilkins A et al., 2003, J of Neuroscience, 23 (12): 4967-4974.

We agree with this last point. The Wilkins et al study is in full agreement with our study as it uses full conditioned medium including EVs and may well describe sEV effects. To specify sEVs as active component versus potential other soluble oligodendrocyte-derived factors, it is essential to compare sEV containing 10 K supernatant with sEV-deprived 100K supernatant, which controls exactly for these soluble factors (so we did). Inclusion of an ND alone condition would show whether soluble factors also have an effect or not, however, this information does not impact the validity of our results regarding sEVs. Although it could strengthen our results, we are convinced that we can skip this experiment for the present study. The essential control is the 100K supernatant and not the ND-alone condition.

We moved Figure 1 (examining the short term effect of sEVs on axonal transport) to the Supplementary Material. Please consider that neurons in culture under full medium conditions are provided with a plethora of factors in favor of all of their growth functions (making it difficult to depict effects of other additional factors). Based on our previously published experiments we actually didn’t expect to see a clear effect of sEVs on AT under full medium conditions and were pleased when detecting the effect on pausing time. Shorter pauses of vesicles during transport means they arrive earlier at their destination (reducing the total duration of transport).

2. Although ultracentrifugation has been widely used to purify exosomes, it has always been criticized for having proteins especially lipoproteins bound or co-pelleted on the outside membrane, such as Li P et al (Theranostics, 2017, Vol 7, 3, 789-804). This issue was not sufficiently addressed in this study. The authors compared the effect of 10K and 100K supernatant and concluded that oligodendrocytes-derived sEV is protective. It is highly likely that a supernatant protein is attached to the outside membrane of sEV and co-pelleted during the 100K spin and be neuroprotective. Additional experiments to rule out this possibility is important to support the authors' claim. Also, why the proteomics and treatment uses different approach to purify exosomes? There is no data presented to indicate that the gradient centrifugation purified sEVs are protective in these assays.

This is an important point. We agree that ultracentrifugation may co-precipitate other factors and we cannot rule out completely that such factors contribute to functional activity (but still would be scientifically relevant). We had addressed this point already in the discussion. However, we cannot find any evidence that this indeed may be the case:

- New Fig. 1D shows that sEVs depicted in density gradients (100 K pellets loaded at bottom and floating up) float in fractions of typical density, while other proteins known to co-purify in 100K pellets and migrating in high density fractions were undetectable (Jeppesen et al., Cell 2019, doi: 10.1016/j.cell.2019.02.029).
- We exclude that factors derived from the medium such as lipoproteins (sEV are collected serum-free) contribute to the observed functional effects. Medium-derived factors co-purifying with sEVs would contaminate both wildtype and mutant sEVs and thus can be excluded to play a major functional role in our study.
- Unfortunately, sEVs collected from density gradients (or centrifuged onto a cushion) have lost functional activity, most likely due to osmotic effects. This a well-described phenomenon in the EV-field (Thery et al., JEV 2018, MISEV guideline update, doi: 10.1080/20013078.2018.1535750). We therefore only applied density-gradient purification for biochemical sEV analysis including the highly sensitive proteomics technique (see page 8, 2^nd^ paragraph).
- Alternatively, we used SEC-isolation of EVs from OL supernatants (Fig. 1 E), which however appears not as efficient in separating sEVs from other non-EV particles and results in large sEV losses in later fractions. When pooled fractions 4-6 were used to treat nutrient-deprived neurons, these SEC-EVs carried the same activity and the neurons were able to maintain their metabolic activity (MTT assay, see below). However, we are convinced that 10 K supernatants present sEVs to target neurons in their closest physiological (native) state and are superior to SEC-isolated EVs, which go through several processing steps including ultrafiltration likely favoring EV-aggregation. We therefore prefer not to use SEC isolated sEVs for laborious axonal transport experiments.

3. The study primarily focus on the axon transport to assess axon health. As the secondary axonal degeneration is the main phenotype in CNP- and PLP-deficient mice, it will be more relevant to test whether sEVs from oligodendrocytes are affecting axon growth, conductivity, and neuronal survival, etc. This is important to rule out the possibility that SNP- and PLP-deficient oligodendrocytes are not secreting toxic sEVs or other factors that negatively affects the survival of neurons and subsequently affects the axon transport. Especially neurons under oxidative stress in Figure 2 showed much reduced axon transport property.

We have studied other functional activities of sEVs, such as survival signaling and electrical activity in previous studies (Fröhlich et al, 2014) showing that sEVs positively affect these parameters. We also performed preliminary experiments on neurite outgrowth and could not detect an effect on neurite outgrowth. However, neurite outgrowth promoting activity would be relevant for development or regeneration, but not necessarily for axonal maintenance were the axon is already formed and needs to be sustained.

We would like to emphasize that axonal transport is actually a very relevant parameter of axonal health and maintenance, as documented in the literature multiple times.

We cannot exclude that mutant-derived sEVs include some factors that negatively affect metabolic activity and axonal transport. The point is that mutant sEVs are clearly different. They may act according to the loss of function or gain of function principles. To further specify the relevant lacking or detrimental factors will be interesting to study in the future. Note added to discussion page 15 (top).

4. The cre-mediated uptake assay of sEV is not convincing. The proteomics results have suggested that the cargos are altered in CNP- and PLP-deficient oligodendrocytes-derived sEVs. Thus, it is very likely that Cre-packaging into sEV is also altered in CNP- and PLP-deficient oligodendrocytes-derived sEVs, contributing to a reduced GFP activation in neurons. However, this may not indicate reduced uptake of sEVs into neurons. These results need to be further confirmed in neurons using additional protein cargos identified in proteomics.

The Cre-assay is used as a means to demonstrate that wild-type and mutant sEVs differ in their efficiency to deliver functional cargo from OL to neurons (more clearly pointed out on page 9, last paragraph). The assay does not give information on the rate of neuronal uptake (we do not state that). Reduced reporter activation in neurons may be due to a defect in sEV composition (qualitative defect, i.e. reduced inclusion of Cre) or the reduced release rate (quantitative dose effect) or a combination of both. In any case, the assay demonstrates that mutant OL-derived sEVs exhibit diminished potency to deliver functional cargo to neurons, when directly compared to wildtype OL-derived sEVs. The strength of the Cre assay is that it provides a readout for the functional activity of the cargo and this assay confirms that on this level the mutant-derived sEVs indeed are less functional.

Minor comments:
1. Figure legend is misleading in describing the ND condition, which makes relevant figures confusing.

Thank you, the figure legend was revised. We think no it is clear and correctly expressed.

2. Immuno-EM should be performed to specifically label MVBs instead of the morphological description.

We do not have at hand a reliable marker specifically labelling MVBs in Immuno-EM. MVBs are readily detected by morphology and MVB identification is highly reliable

3. Information missing as to the number of sections or mice examined for MVB quantification.
Number of mice n=3 (individual data points in figure, also pointed out in legend), 10 sections per mice. Clarified in materials and methods, page 22, 2^nd^ paragraph.


**Reviewer 3**: Tomas Falzone – note that this reviewer has waived anonymity
This work entitled "Oligodendrocyte-derived exosomes promote axonal transport and axonal long-term Maintenance" by Frühbeis C, et al. is focused on understanding the possible role of extracellular vesicles released by oligopdendrocites in the regulation of transport dynamics and in long term neuronal survival. This question has significant relevance in the process of neurodegeneration and regeneration. To lay out this proposition Frühbeis uses primary neuronal cultures transfected with a fluorescent cargo (BDNF) and incubate them with extracellular vesicles obtained from oligodendrocytes (O-EVs). This gave mild effects on reducing the pausing time of BDNF. Then authors showed that O-EVs were able to prevent some transport defects induced by oxidative stress or nutrient deprivation. Authors also studied how two mutant mouse lines (PLP- and CNP-deficient mice), which are established mouse models of glia-dependent secondary axonal degeneration, affect the production of O-EVs.
The quantity of O-EVs release and the protein conformation within released O-EVS is impaired in these mutant mice. Downrgulation and upregulatin of protein content within O-EVs was determined by mass-spec and candidates were validated by western blot. In addition, mutant O-EVs failed to deliver CRE enzyme activity in a functional test Finally authors perform experiments to show that WT O-EVs are sufficient to restore axonal transport from nutrient deprived conditions, however EVs obtained from mutant cells do not produce this effect. The proposition that normal O-EVs regulate axonal transport properties is novel and very interesting. Also that mutant O-EVs lack key protein components and fail to promote transport under nutrient deprived conditions. However, I see within the manuscript two sets of non-cohesive group of experiments and many experimental settings and statistical conflicts that need to be solved before considering this work for publication.

Thank you for acknowledging the novelty and significance of our study and for important input regarding the axonal transport experiments. We reevaluated the data considering your points.

Major concerns
* The title does not reflect the relevance of exosome variations in mutant mice. The main problem is that the main questions, regarding transport dependence on O-EVs and defects in mutant O-EVs release are not well resolved. This work would be greatly improved with more cohesiveness regarding both controls and experimental design. As well as a clear focus in answering the most relevant questions that authors themselves presented.

We changed the title according to the suggestion to reflect impaired exosome release in mutant mice, which indeed is the most relevant aspect in the study. We understand that the different experimental protocols applied in axonal transport experiments cause confusion and therefore focused the main manuscript on the nutrient deprivation experiments and the comparison between wild-type and mutant oligodendrocyte-derived sEVs.

* Experimental setting should be standardized in order to compare different results in different figures properly. Treatment time frames differ from Fig 1 (30 min) of O-EVs incubation to Fig 2-3 (15 hours), this should be explained.

With focus on nutrient deprivation, those experiments delivering the main message are standardized. The short treatment with sEVs (30 min) of unstressed neurons was initially designed to analyze the immediate effect on axonal transport. As we still think this is an important result, but understand that this harms the coherence of the manuscript, we moved the data to the Supplementary Material (as well as the oxidative stress data, formerly Fig. 2, see also explanation page 5, 2^nd^ paragraph).

* In fig 1, authors showed: "Primary hippocampal neurons expressing BDNF-mCherry were exposed to oligodendrocyte-derived sEVs". In Fig 3 authors showed :"Primary hippocampal neurons were treated with sEV-conditioned oligodendrocyte supernatant (10K) or sEV-deprived supernatant (100K) as control". This change in methodology and the possibility that affect neurons differently should be discussed mainly due to the changes that are observed in one experiment compared to the other.

Figure 1 now moved to Supplementary Material. To use the 10K and 100K supernatant actually reflects the evolution of the experimental design. Pelleting sEVs by 100.000 x g ultracentrifugation may result in sEV aggregation affecting their functional competence. Therefore, we moved to 10K supernatants were sEVs are in a “native” state and included 100K supernatants as control. This is now clearly evolved and explained in the manuscript (see page 6).

* Imaging experiment in electroporated primary hippocampal neurons were performed and imaged at: "at DIV 2 or DIV 3 after isolation". This raises a significant concern about whether the authors are truly observing axonal transport or they are just looking at intracellular dynamics in undefined projections. It has been thoroughly demonstrated that axon determination start between day 2 to 4 in culture of hippocampal neurons (Dotti, journal of Neuroscience 1988). Method for imaging are incomplete. How authors defined the axon? Where in the axon between cell body and axonal tip imaging was generated? Was microtubule polarity defined in order to identify the axon projection and maturation?

We identified axons based on the Dotti work. The imaged axons were selected according to a defined and well standardized morphological aspect: axons are 1,5 times longer than the longest neurite. The images were generated between 3 to 5 µm far away from the cell body and 3 to 5 µm before the axonal growth cone. We apologize that we forgot to provide these details and added this information to the methods (page 20).

* Why authors performed time lapse movies every 3 seconds to register a fast moving vesicle? In many of the kymographs showed in figures it is very difficult to follow the vesicle trajectory.

This was due technical prerequisites in the pilot experiments (now in Supplementary Figures): camera sensitivity and the bleaching of the mCherry fluorophore did not allow shorter frames. We agree that the resolution is not perfect, but dynamics can still be observed and the relevant parameters can be measured. Imaging of nutrient deprived neurons was performed with a more sensitive system (0,5 s per frame).

* A major concerns is placed in the identification of 40 um/sec velocities or above. Since authors performed time lapse movies every 3 seconds particles should disappear from view of field in this time frame. Some velocity conversion does not fit with previous velocities described for BDNF that range at around 1 um/second (Olenick, Journal of cell Biology 2019). Not to even mention that 80 um/sec velocity for a vesicle has not been described in bibliography so far.

Thank you for this remark, which brought to our attention a mistake on our side (we apologize for that). The correct unit for velocity in the graphs is µm/min. In DIV 3 to 6 rat cortical neurons, BDNF-mcherry containing vesicles move anterogradely in axons at 2.5 µm/sec and retrogradely at 2 µm/sec (Zala et al, Cell 2013). This means the velocity we measure is in the published range (Fig. 2D). We also added scale bars for time and distance to the kymographs.

* We have noticed that serum deprivation reduces movement proportion and increases pauses, but accelerate velocities? This non expected increase in velocities should be further addressed and discussed.

We agree. This can be explained by the possibility that 100K supernatants contain other oligodendrocyte-derived soluble factors that influence the velocity of anterograde vesicles. Reflected on page 6, bottom.

* The number of neurons analyzed in each experiment is too small for axonal transport experiments with high variability and with not certain definitions of projections analyzed.

Projections are defined according to morphological criteria (see above). We consider the number of neurons sufficient to obtain a body of data that can be well interpreted by the reader and exhibit statistic significance.

* In general, imaging methods are poor. Authors should state how pausing has been defined and how pausing time was calculated. In Figure 1 authors should state the n of particles analyzed and the number of pauses. Figure legends in general should be improved
A pause was defined as a period ≥10 s, during which the vesicles move less than 5 μm. The cutoff of 5 μm was chosen based on the average diameter of vesicles, which was approximately 1 μm. Pausing time is the percentage of time that a vesicle spends pausing over its total time movement (defined in methods). Modified from Luke AD Bury and Shasta L Sabo, Neural Dev. 2011; 6: 24. Up to 50 particles per Kymograph were analyzed (added to legend).

* As it is observed from graphs, velocities are not distributed with a normal distribution. Have the authors used a test for normality? Why data is not analyzed as non-parametric data?

Thank you for bringing this to our attention. We reanalyzed all data using different statistic tests and were pleased to see that the outcome is only marginally different (regarding p-values) and does not alter the general statements made and the data interpretation. We finally used Shapioro Wilk test to test for normality (some data indeed were not normally distributed and decided to run a non-parametric unpaired Kruskal-Wallis test followed by Dunns multiple comparison test (Figure 2, 6, and Supplementary Figure 2) or Mann-Whitney test (Supplementary Figure 1).

* Why do authors use HEK293T cells as control for some experiments and astrocytes for others? HEK293T cells are human derived, since primary cultures are from mice, this cell line is not the best control. The use of a mouse derived cell line or primary culture (like astrocytes) is a better option. Furthermore, it would be interesting to see how exosomes from primary astrocytes affect WT neurons in standard conditions as a control (in comparison to Oligodendrocyte exosomes).

We started out in the first set of experiments using HEK293-derived sEVs as control (for another type of EV) and then proceeded to astrocyte-derived EVs. We agree that this is a perfect control, but actually would not have been surprised if they carried a similar activity as OL-derived EVs. We were happy to see that this perfect control finally worked out. This will be less confusing now, as the oxidative stress experiment has also been moved to the Supplementary Material (Fig. S2).

* Despite CNP and PLP being mostly expressed in Shawn cells and oligodendrocytes (there is evidence of PLP expression in neurons (doi: 10.1002/jnr.22121)), it would be interesting to determine neuron mutant morphology and axonal transport and, if changes arise, the effect of WT EVs over these mutant neurons.

Thank you for the suggestion, we principally agree. However, this is out of the scope of this study. The effect of PLP-deficient oligodendrocytes on axonal transport has also been observed in a transplantation setting, were neurons did not carry the mutation. This indicates that not the minor expression of PLP in neurons (which by the way appears soma-restricted and therefore is called sr-PLP) is responsible for the observed effect.

* It is not clear how mislocalization of MVB in mielinated tracks is correlated with abnormal exosome release by oligodencrocites since localization is differentially impaired between mutants, but both mutants showed reduced proteomic contents in O-EVs.

We apologize, if this was not clearly pointed out in the manuscript. MVB localization and sorting of EV-components reflect two distinct mechanistic defects in the ko-cell leading to quantitative and qualitative impairment of EV release. The quantitative defect can be explained by MVB mislocalization leading to a reduced rate of EV-release (PLP-ko exhibits MVB accumulation and CNP-ko in MVBs are localized away from MVB release site). The observed qualitative defect suggests aberrant sorting of exosomal proteins in mutant cells or that MVB subpopulations maybe differently affected (see comment Reviewer 1, see discussion page 14).

* Why do authors used a Student t tests instead of One Way ANOVA and a post-test for multiple comparisons?

This refers to MTT tests in Fig. 5, rigtht? After testing for normality, two data sets were directly compared using t-test.

* In discussion, authors state that the findings of this work proved that EVs have a long term effect on neurons, however experiments were not carried over periods longer than 24 hs.

24 h is a long time for a cell in a culture dish. The phenotype of mutant mice develops later in life and reflects in our view a long-term deprivation of support by oligodendroglial sEVs.

* Since it has been proven that EVs modulate neurons metabolic rates, electrophysiology and now axonal transport, a discussion regarding axonal transport modulation being a direct effect of EVs or a secondary effect, subsequent to metabolic or electrophysiological changes should be included

See discussion page 13, 1s para, second-last sentence.

Minor concerns:
* In introduction citations are missing. Furthermore, regarding to axonal transport.

More citations included

* -What are red arrows pointing at?

The red arrows point to the same vesicle that can be follow throughout the different frames. Explained in legend.

* -Bar for distance and time frames are missing in figures.

Added.

* Are kymographs representative of the pictures showing time lapse progresion in the manuscript?
Yes


**Reviewer 4:**
Kramer-Albers and coworkers previously showed that small EVs (exosomes) released by oligodendrocytes sustain neuron metabolism , especially under stress conditions. In this study they build on these previous findings and show that oligodendrocytes-derived sEVs promote axonal transport, a process that is essential for axonal integrity. In addition, they show that exosome production is quantitatively and qualitative impaired in oligodendrocytes from PrP- and CNP-ko mice, which exhibit secondary axonal degeneration.

Collectively, these novel data suggest that impaired exosome production from oligodendrocytes is responsible for alterations of axonal transport and consequent axonal degeneration in the mutant mice. By suggesting the mechanism underlying secondary axonal degeneration in PrP- and CNP-ko transgenic mice this excellent work has a high impact in the neurodegeneration field and provides a significant advancement in current understanding of how glial cells dysfunction contribute to axonal degeneration. In general the manuscript is well written, the experiments are well done and clearly described and appropriate controls are include
Thank for the positive evaluation of this study and our work.

However, there are a few points that need to further explored or clarified

-While fig 1 shows the effects of short treatment with sEVs on axonal transport in control conditions, the effects of prolong sEV treatment (12-13 h) without H202 is not reported in fig 2. This makes impossible to verify whether or not Ols-derived sEVs completely or partially rescue the alterations of intra-axonal transport caused by stress and to appreciate the transport defects induced by H202. Does prolong treatment influence basal transport kinetics besides decreasing the pausing time? How prolong treatment influences vesicle transport (anterograde and retrograde transport and the percentage of static EVs….) in the absence of stressful stimuli should be included in fig 2. The same hold true for fig3 where the effect of treatment with sEV-conditioned medium in the absence of nutrient deprivation is undefined. In order to conclude that sEVs preferentially protect axons under stressful conditions, the action of sEVs /conditioned medium containing sEVs in control conditions
should be shown.

We principally agree with these points. Our first pilot experiments (former Figure 1, now Supplementary Figure 1) were designed to see the immediate short-term effect of sEVs on axonal transport. Seeing the results, we were convinced that under full medium conditions and prolonged treatment, the effect of sEVs would not be visible. The neurons in full medium culture are provided with an excessive supply of nutrients and protectants most likely masking the potential effect of a single factor such as sEV (think about addition of a single growth factor in the presence of serum to a culture: it is impossible to depict an effect of the growth factor under these conditions after longer incubation, maybe only after short term incubation, when looking at the signaling pathway). We expect that long term treatment with sEVs under full medium conditions would result in no difference between treatment and control. Therefore, we concentrated our experiments on the stress conditions. Although we intended to demonstrate this clearly and planned to add the full medium condition to the manuscript, we unfortunately were not able to conduct these experiments in the last months (COVID19 lockdown). We apologize and would like to bring to attention that the relevant controls are included in the manuscript (as also mentioned by this reviewer and other reviewers). We also focused the manuscript on the significant and most coherent data set, which we think underscores the main message and will avoid confusion of different treatment conditions.

- Why sEVs were resuspended in PBS before addition to neurons, given the cells were live imaged at 37°c and 5% CO2, thus likely in neuronal medium? Medium dilution with PBS may cause neuronal damage.

This is a standard procedure in our lab (we made good experience in the lab with resuspending EVs in PBS as supported by NTA measurements and other experiments). Addition of a relatively small volume of PBS to the neuronal culture medium does not harm the neurons. In the nutrient deprivation experiments, EVs were harvested and applied to neurons in the correct neuronal growth medium.

-It is not clear to me from the "sEV treatment and stress condition…" paragraph in the methods whether neurons were exposed to similar concentration of sEVs produced by HEK293T or astrocytes compared to oligodendrocytes. The number of particles produced by HEK293T and astrocytes should be directly estimated by NTA analysis.

We agree that NTA measurement would be the best way to normalize for. However, NTA is not performed within minutes, rather hours, and the workflow did not allow for this time to be included. Astrocytes and HEK cells are much larger cells as compared to oligodendrocytes, likely secreting more sEVs per cell. We therefore normalized sEVs derived from these cells by the volume of supernatant harvested from a confluent culture covering the same area. See information in methods on page 19, bottom).

-For clarity, define sEV-conditioned oligodendrocyte supernatant (10k) the first time it is mentioned in the results. To help the reader, make clear that the 10k supernatant is the sup after centrifugation of large EVs.

Thank you. Clarified on page 5, 6 and Fig. 1A.

-Figure 4B-D, G, Fig 5 F Please report in the figs or in the legend whether changes in MVB distribution are statistically significant or not.

The number of biological replicates is n=3 mice. As recommended by the Graph Pad Prism software, we performed statistics only on n > 4 for producing reliable values. When performing statistics (non-parametric test), most p-values are not significant (>p=0.5). We prefer to stick to that practice and limit reporting to showing SEM and individual data points, which is transparent for the reader.

-Page 8, second line. Please clarify why the biological process "transport" fits with the biological activity of small EVs for readers not in the EV field
Clarified on page 8, end of second paragraph.

- Page 9. The authors refer to metabolic activity/cell viability of neurons without describing which functional parameter/assay they were measuring. Please make clear in the results that neuronal metabolism/integrity was evaluated by the MTT assay.
Clarified on page 10

-With respect to neuron metabolism, the MTT assay is not enough to claim that EVs support the metabolic activity of neurons (as repetitively reported in the text, see for example the discussion at page 11) . Complementary approaches (e.g. Seahorse evaluation of oxygen consumption rate (OCR) and extracellular acidification rate) should be used to better define how sEVs influence neuronal metabolism. This would significantly strengthen the results of the study.
Thank you for the suggestion. The MTT assay measures the reduction potential reflecting metabolic competence. While we agree that the seahorse assay may strengthen our results, we think that the MTT assay is adequate to make the main claims at this point (PLP and CNP-ko sEVs lack functional activity). We tried to use the term metabolic activity more carefully (e.g. abstract).

- Please clarify why cell viability was analyzed in cortical neurons while vesicle transport was explored in hippocampal cultures. It should be reported in the results, not only in the methods, that axonal transport was analyzed in immature, developing neurons. Moreover, the differentiation stage of neurons assayed by MTT has not been specified. Please add this info in to the text

Hippocampal neurons can be cultured in low density, required for imaging the axons (not possible with cortical neurons). The neurons are transfected before seeding and therefore the neurons have to imaged early, as soon as the axon can be differentiated from dendrites. Explained in results.
