# Supplementary material for: Oligodendrocytes support axonal transport and maintenance via exosome secretion
Source: PLoS Biol. 2020 Dec 22;18(12):e3000621. doi: 10.1371/journal.pbio.3000621 (PMC7787684; doi:10.1371/journal.pbio.3000621)
Supplement: Attachment — Submitted filename: Reviewers Response_Rev2_final .docx [file pbio.3000621.s015.docx]

Dear Dr Krämer-Albers,

Thank you very much for submitting a revised version of your manuscript "Exosomes derived from PLP- and CNP-deficient oligodendrocytes lack the ability to support neurons and fail to promote axonal transport" for consideration as a Research Article at PLOS Biology. Thank you also for your patience as we completed our editorial process, and please accept my apologies for the delay in providing you with our decision. This revised version of your manuscript has been evaluated by the PLOS Biology editors, the Academic Editor and three of the original reviewers.

The reviews are attached below. You will see that the reviewers find the manuscript much improved and that they think most of their concerns are now resolved. Nevertheless, Reviewer 2 raises some points that should be addressed either by the proposed immunobead exosome depletion experiment or by specifically stating in the discussion that the possibility that some of the effects observed are mediated by a factor co-purified with the exosomes cannot be categorically ruled out. Reviewer 3 would like you to clarify whether or not the same data are being presented more than once in Figures 2 and 6 – if this is the case, it should be fixed - and other points that can be addressed by improving the description of the methods and re-analyzing some of the data.

After discussing the reviews with the academic editor, we are pleased to offer you the opportunity to address the points raised by the reviewers in a revised version that we anticipate should not take you very long. We will then assess your revised manuscript and your response to the reviewers' comments.

We expect to receive your revised manuscript within 1 month.

Reviewers’ comments

**Rev. 2:**
In this revised manuscript, the authors have made efforts to include new data to support the original conclusions. While the COVID pandemic has made it difficult to perform additional experiments to fully address my concerns, the lack of such experiments pose substantial ambiguity in whether it is sEV or other soluble factors that mediate oligodendroglial support to axons.

Thank you for acknowledging our efforts. We agree that we cannot completely rule out the contribution of factors that co-precipitate with the sEVs in the 100,000 x g centrifugation (we thought we had expressed this in the discussion before). We amended the discussion to emphasize this further (page 12).

While the authors emphasize the comparison between 10k supernatant (+ exosome) and 100k supernatant ( -exosome), the ultracentrifugation is unable to separate sEV and other proteins that attach on sEV. The similar density distribution of sEV as described in previous studies does not support whether there are other proteins associated with the 100k pellet, as it depends on associated protein quantity. It would be imperative to test whether supernatant from immunobeads depleted exosomes still give functional phenotypes or not, the diminished effect from supernatant would add more convincing data to support the observed axon fast transport is indeed mediated by exosomes, but not associated other soluble factors.

We agree and are fully aware of this. However, immunobead depletion is more complex than anticipated and will finally not resolve the issue completely for the following reasons:

1. Soluble factors or other particles may co-precipitate in similar non-specific way (e.g. by binding to the bead matrix).
2. The functionally active sEV subpopulation may not carry the epitope targeted by the immunobeads and thus not removed.

Indeed, the often targeted tetraspanin CD63 is only weakly expressed in oligodendrocytes (see <https://www.brainrnaseq.org/>) and a considerable fraction of oligodendroglial sEVs may also lack CD81 or CD9. An in-depth analysis of the relevant component in the 10K supernatant and the potential sEV subpopulations carrying the functional activity is underway and will be subject of a future report.

Therefore, we decided to further discuss this topic (see page 12, bottom):

*“Based on the present results we cannot completely rule out that a minor fraction of other sEVs, non-EV particles, or soluble factors contribute to the reported functional activity of oligodendroglial sEVs..................However, it cannot be entirely excluded that soluble factors associating with sEVs in the 100K pellet contribute to the observed effects. Further studies such as immuno-depletion of specific sEV-populations are required to identify the relevant sEV subpopulation and to finally specify the active component within the 10K supernatant.”*

In addition, the authors observed loss of functional mutant sEV data in axon transport. However, given the secondary axon degeneration phenotype in both mutant mice, the effect on reduced axon transport by mutant oligo-derived sEV remains insufficient to conclude that this would be the underlying mechanisms for axon degeneration. It should be demonstrated whether mutant oligodendrocytes derived sEV has potential direct toxicity to axons, which may directly link to the axon degeneration phenotype.

It is well possible that mutant sEVs also actively contribute to axon degeneration. This is expressed in the discussion (page 15, 1st paragraph, last sentence): “*Moreover, it should be noted that proteins upregulated in PLP- and CNP-null sEVs may exhibit detrimental effects on receiving neurons and impair axonal transport.”*

However, in the metabolic activity and axonal transport assays, mutant sEV treated neurons perform on a similar level as neurons treated 10K supernatants depleted from wild-type EVs rather indicating a lack of activity (loss of function). There is presently no direct evidence for a detrimental action of mutant sEVs and its contribution to axonal degeneration. Importantly, our main conclusion that impaired sEV-release in PLP- and CNP-mutant mice provides an explanation for the secondary axonal degeneration in these mice is not affected by these assumptions.

In my opinion, both issues are directly related to the main conclusion of the current manuscript. It is thus important to clarify these key questions. While the revised manuscript provided explanation on prior critiques, the lack of these experimental results makes it frail in interpreting key observations and undermines the novelty of the current study.
We are optimistic that we were able to clarify the remaining concerns. We are convinced that our study provides compelling evidence for an important role of exosomes in neuron-glia interaction and glial support contributing to the understanding of long-term axonal maintenance.


**Rev. 3:**
I appreciate the response and the efforts invested by the authors in answering reviewers' concerns and also understand the difficulties arising due to worldwide situation. I`m pleased to see that authors decided to adjusted title to findings, focused the work and made it more cohesive. I consider the work very relevant but would like to re-state some concerns that have not been properly addressed.

We highly appreciate your efforts and thank you for providing us with a critical reflection and constructive suggestions, which lead to substantial improvement of the manuscript.

I consider that data duplication in figures should not be presented. It seems that what was previously shown as figure 7, it is now shown duplicated partially as Figure 2 and repeated again in Figure 6?.
It could be that I`m making a mistake in identifying this, and I`m sorry if what I`m saying is not correct. But in case it is, should not be presented this way.

That is actually right. For reasons of comprehension, we reproduced data from Fig. 2 C-E in Fig. 6 C-E. We consider this essential as a reference, since otherwise the axonal transport promoting activity lacking in the mutant sEVs would not be visible in the graphs. We apologize for not having clearly indicated this before.

We clarified this now in the legend of Fig. 6 (page 31): “*Data from control, ND (+100K SN) and ND+WT EVs are reproduced from figure 2 as reference.”*

I appreciate the clarifications included in methods for the identification of axons and cited seminal papers. As authors realized, axons at day 2 to 3 after neuronal plating are in their determination phase being only 1,5 longer than the longest dendrite. So, a statement is necessary to be included in text clarifying the early maturation stage of neurons.
The text was edited accordingly:

- Results (page 5, 1^st^ paragraph): *“We performed live imaging in developing primary hippocampal neurons.....,*
- Methods (page 20, second last paragraph): *“Imaged neurons were in an early maturation stage representing stage 3 of axon determination.”*

I`m relief that velocities observed are now in literature range and would suggest to express them as um/sec to standardize it with most of the work on transport dynamics. It is still confusing whether authors are looking to segmental velocities or average velocities. And even, are plotted velocities correspond to average particle velocity or to kymograph average velocity?, since the procedure for this calculation is still not included in methods.

We measured average velocities of single vesicles. The average velocity is calculated as (final distance - initial distance)/ (final time - initial time) of each vesicle trace.

We clarified this in the methods (transition page 20/21)*: “Average velocities of single vesicles were calculated as (final distance - initial distance)/ (final time - initial time) of each vesicle trace. Vesicles with a mean velocity below 5 µm/min (0,083 µm/sec) were considered static.”*

With respect to pauses, my advice is considering pauses to no movement within moving particles. As per your stated criteria a particle moving at lower than 0,5 microns per second is considered pausing but your average speed in control conditions is near 0,6 um/sec.

Pauses were assessed within moving particles (see page 21 top: “*A pause was defined as a period ≥10 s, during which the vesicle moves less than 5 μm”*). Since pauses contribute to the overall average velocity, the mean velocity may be slow and close to the defined speed of 0.5 µm/sec.

I`m pleased that result differences are maintained after performing statistical test for non parametric data when it is required. I would suggest that data without normality is presented as median, boxes and whiskers, instead of average and errors.

We agree. We expressed that data as suggested (see Fig. 2 C-E, Fig. 6 C-E, Fig. S1 C-E, Fig. S2 C-E).


Rev. 4:
The authors satisfactorily addressed all the issues raised in my previous review.

We thank the editors and all reviewers for a fair and constructive evaluation process and their valuable efforts in improving the manuscript.
