# Supplementary material for: Oligodendrocytes support axonal transport and maintenance via exosome secretion
Source: PLoS Biol. 2020 Dec 22;18(12):e3000621. doi: 10.1371/journal.pbio.3000621 (PMC7787684; doi:10.1371/journal.pbio.3000621)
Supplement: Attachment — Submitted filename: Response to Editor_R3_final .docx [file pbio.3000621.s016.docx]

Dear Dr Krämer-Albers,

Thank you for submitting your revised Research Article entitled "Exosomes derived from PLP- and CNP-deficient oligodendrocytes lack the ability to support neurons and fail to promote axonal transport" for publication in PLOS Biology. I have now obtained advice from the Academic Editor and have discussed the comments with the team of editors.

We're delighted to let you know that we're now editorially satisfied with your manuscript. However, we would like you to include in the manuscript the clarification you recently sent us on the point regarding the replication data in Fig. 2 and 6. In addition, please consider our suggestion to improve the title and make it more accessible for a broad audience as follows:

"Oligodendrocyte genetic defects impair the secretion of glia exosomes and their ability to support nutrient-deprived neurons and promote axonal transport"

Before we can formally accept your paper and consider it "in press", we also need to ensure that your article conforms to our guidelines. A member of our team will be in touch shortly with a set of requests. As we can't proceed until these requirements are met, your swift response will help prevent delays to publication. Please also make sure to address the data and other policy-related requests noted at the end of this email.

To submit your revision, please go to https://www.editorialmanager.com/pbiology/ and log in as an Author. Click the link labelled 'Submissions Needing Revision' to find your submission record. Your revised submission must include the following:

- a cover letter that should detail your responses to any editorial requests, if applicable

- a Response to Reviewers file that provides a detailed response to the reviewers' comments (if applicable)

- a track-changes file indicating any changes that you have made to the manuscript.

------------------------------------------------------------------------

DATA POLICY: IMPORTANT - PLEASE READ

Thank you for complying with our data policy and sending us the data underlying all individual quantitative observations summarized in the figures. However, we are still missing data from the following figures that you will need to provide:

Fig. 3E; Fig. 4A, B; Fig. S3C-D and Fig. S5

NOTE: the numerical data provided should include all replicates AND the way in which the plotted mean and errors were derived (it should not present only the mean/average values).

Please also ensure that figure legends in your manuscript include information on WHERE THE UNDERLYING DATA CAN BE FOUND, and ensure your supplemental data file/s has a legend.

Please ensure that your Data Statement in the submission system accurately describes where your data can be found.

Please revise the Data Availability statements that you have included in the paper (“The data that support the findings of this study that are not included within the paper and its supplementary information files are available from the corresponding author upon reasonable request”), as it contradicts the statement reflected in the metadata section (“All relevant data are within the paper and its Supporting Information files”). All relevant data should be made available according to our policy.

Dear Dr. Alvarez-Garcia,

Thank you and the Academic Editor for your work, we are pleased and look forward finalizing the publication process. The following changes were made to the manuscript:

- A statement regarding data replication between Fig 2 and Fig 6 and clarification that each experimental setup comprised all treatment conditions was included in the manuscript (page 10) and is also mentioned in the Figure legend of Fig 6.
- We appreciate your suggestion to improve the title regarding accessibility to a broad readership. Despite the proposed title would be fine for us, we would like to bring it one step further. We prefer a shorter title **“Oligodendrocytes support axonal transport and integrity via exosome secretion”,** which in our opinion brings our study to the point However, if you feel this title is too broad, we alternatively wish to choose: “Glial genetic defects affecting axonal integrity impair the secretion of oligodendrocyte exosomes and their ability to promote axonal transport”, which reflects a slight modification of your suggestion. We think it is important to deliver in the title the relationship between the axonal phenotype of the mutants and the functionality of glial exosomes (actually, I uploaded a similar sentence now as a blurb, which fits perfectly together with the title).
- The Data availability statement was revised and the missing original data were added to the Data files of the Supplementary Material (Fig. 3E and S3C-D can be found in S1 Data and Fig. 4A, B and Fig. S5 in S2 Data). Information regarding the location of underlying data was added to the figure legends.
- Supplementary Material was submitted as individual files and captions were added to the manuscript file.
- A few minor changes to the text in sake of correctness (see marked manuscript, page 6, 10/11, 14)

Please do not hesitate to contact me with any question or further comment.

Kind regards

Eva-Maria Krämer-Albers
